# Ribonuclease L and metal-ion–independent endoribonuclease cleavage sites in host and viral RNAs

**DOI:** 10.1093/nar/gku118

**Published:** 2014-02-05

**Authors:** Daphne A. Cooper, Babal K. Jha, Robert H. Silverman, Jay R. Hesselberth, David J. Barton

**Affiliations:** ^1^Department of Microbiology, University of Colorado School of Medicine, Aurora, CO 80045, USA, ^2^Department of Cancer Biology, Lerner Research Institute, The Cleveland Clinic, Cleveland, OH 44195, USA, ^3^Department of Biochemistry and Molecular Genetics, University of Colorado School of Medicine, Aurora, CO 80045, USA and ^4^Program in Molecular Biology, University of Colorado School of Medicine, Aurora, CO 80045, USA

## Abstract

Ribonuclease L (RNase L) is a metal-ion–independent endoribonuclease associated with antiviral and antibacterial defense, cancer and lifespan. Despite the biological significance of RNase L, the RNAs cleaved by this enzyme are poorly defined. In this study, we used deep sequencing methods to reveal the frequency and location of RNase L cleavage sites within host and viral RNAs. To make cDNA libraries, we exploited the 2′, 3′-cyclic phosphate at the end of RNA fragments produced by RNase L and other metal-ion–independent endoribonucleases. We optimized and validated 2′, 3′-cyclic phosphate cDNA synthesis and Illumina sequencing methods using viral RNAs cleaved with purified RNase L, viral RNAs cleaved with purified RNase A and RNA from uninfected and poliovirus-infected HeLa cells. Using these methods, we identified (i) discrete regions of hepatitis C virus and poliovirus RNA genomes that were profoundly susceptible to RNase L and other single-strand specific endoribonucleases, (ii) RNase L-dependent and RNase L-independent cleavage sites within ribosomal RNAs (rRNAs) and (iii) 2′, 3′-cyclic phosphates at the ends of 5S rRNA and U6 snRNA. Monitoring the frequency and location of metal-ion–independent endoribonuclease cleavage sites within host and viral RNAs reveals, in part, how these enzymes contribute to health and disease.

## INTRODUCTION

Endoribonucleases include metal-ion–dependent enzymes and metal-ion–independent enzymes (1). Metal-ion–dependent enzymes (e.g. RNase P and RNase MRP) cleave single- and double-stranded RNA to produce RNA fragments with 5′-phosphate and 3′-hydroxyl termini. In contrast, metal-ion–independent enzymes (e.g. RNase L, RNase A and IRE1) target single-stranded RNA exclusively, producing RNA fragments with 5′-hydroxyl and 2′, 3′-cyclic phosphate termini (Table 1). The specificity of metal-ion–independent endoribonucleases for single-stranded sites in RNA is due in large part to the mechanism of RNA cleavage. A structurally flexible 2′-OH, in the context of single-stranded RNA, functions as the nucleophile, resulting in the generation of the 2′, 3′-cyclic phosphate at the site of RNA cleavage by metal-ion–independent endoribonucleases.

**Table 1.** Endoribonucleases^a^

Enzyme	Biological role(s)	2′, 3′-Cyclic phosphate^b^	Citations
RNase L	Antiviral response	Yes	Zhou *et al.* (2)
RNase A (RNase1-8, Angiogenin)	RNA degradation, Host response, tiRNA stress response	Yes	Yang (1)
			Tuck and Tollervey, (3)
			Yamasaki *et al.* (4)
RNase T2	RNA degradation	Yes	Luhtala and Parker, (5)
C16orf57/hUSB1	U6 snRNA maturation	Yes	Mroczek *et al.* (6)
			Shchepachev *et al.* (7,8)
IRE1	ER stress response, Antibacterial defense	Yes	Sidrauski and Walter, (9)
			Cho *et al.* (10)
PP1	snoRNA maturation	Yes	Laneve *et al.* (11)
RNase P	tRNA maturation	No	Esakova and Krasilnikov, (12)
RNase MRP	rRNA maturation	No	Esakova and Krasilnikov, (12)
DOM34	No-Go mRNA decay, rRNA decay	Unknown	Lee *et al.* (13)
			Cole *et al.* (14)
G3BP, SMG6, and others	Stress granules and others	Unknown	Schoenberg (15)
NendoU	Coronavirus and arterivirus replication	Yes	Ivanov *et al.* (16) Nedialkova *et al.* (17)

^a^Metal-ion–dependent and metal-ion–independent nucleases reviewed by Yang (1).

^b^Metal-ion–independent endoribonucleases produce RNA fragments with 2′, 3′-cyclic phosphates, whereas metal-ion–dependent enzymes do not.

Ribonuclease L (RNase L) is a well-studied endoribonuclease associated with lifespan (18), cancer (19,20), antibacterial defense (21) and antiviral defense (2,22,23). Based on its homology with IRE1 (24), a metal-ion–independent enzyme that produces RNA fragments with 2′, 3′-cyclic phosphate termini (1,25), RNase L is expected to produce RNA fragments with 2′, 3′-cyclic phosphate termini (Table 1). RNase L cleaves host and viral RNAs predominantly at single-stranded UA and UU dinucleotides (26), although the structural context of UA and UU dinucleotides in RNA greatly impacts the magnitudes of cleavage by RNase L (27). Host and viral RNAs cleaved by RNase L function as ligands for RIG-I and MDA5 (28,29), amplifying type I interferon expression and antiviral responses. Several viruses encode mechanisms to directly counteract RNase L. Group C enteroviruses encode an RNA structure that functions as a competitive inhibitor of RNase L (30–33), whereas certain strains of coronavirus and rotavirus express a 2′, 5′-phosphodiesterase that destroys 2′, 5′-oligoadenylates [2-5A or p_x_5’A(2’p5’A)_n_; x = 1-3; n ≥ 2] (34,35), the allosteric activator of RNase L. Recently, the L* protein of Theiler’s virus was shown to directly inhibit RNase L activity (36). Characteristic RNase L-dependent (37) and RNase L-independent (38) cleavage patterns are evident in ribosomal RNAs (rRNAs) during virus infections; however, the precise cleavage sites in rRNAs have not been defined.

Hepatitis C virus (HCV), which is restricted by the 2-5A/RNase L pathway in tissue culture cells (39), is able to overcome both innate and acquired immune responses in patients to establish persistent infections. Nonetheless, RNase L is predicted to influence the outcomes of HCV infections (40), and RNase L cleavage sites in HCV RNA have been mapped by primer extension (27).

To detect and quantify RNase L cleavage sites in host and viral RNAs, we optimized methods using *Arabidopsis thaliana* tRNA ligase, an enzyme that specifically recognizes RNA fragments with 2′, 3′-cyclic phosphate termini (41). We validated the methods using defined viral RNAs cleaved with purified RNase L and RNase A, as well as RNA from uninfected- and poliovirus (PV)-infected HeLa cells under conditions where RNase L was activated during the course of the virus infection (30). As described herein, we discovered discrete regions in HCV and PV RNA genomes that were profoundly susceptible to single-strand–specific endoribonucleases, as well as regions that were largely resistant to cleavage by single-strand–specific endoribonucleases. We identified a constellation of RNase L-dependent and RNase L-independent cleavage sites in rRNAs and we mapped these cleavage sites onto the secondary and tertiary structures of the human 80S ribosome (42). In addition, we detected 2′, 3′-cyclic phosphates at the end of U6 snRNA, consistent with the enzymatic activity of C16orf57 (6,7). Lastly, and unexpectedly, our results suggest that 2′, 3′-cyclic phosphates are present at the end of 5S rRNA.

## MATERIALS AND METHODS

### PV and HCV RNAs

PV (Type 1 Mahoney) and HCV (genotype 1a) RNAs were used in this study. A plasmid encoding PV cDNA [pT7-PV1(A)_80_] was generously provided by James B. Flanegan (University of Florida) (43). A plasmid encoding HCV cDNA (P90/HCVFLlong pU) was generously provided by Charlie Rice (Rockefeller University) (44). PV and HCV RNAs were synthesized from linearized cDNA clones using T7 polymerase (Epicentre), as previously described (27,30).

### Viral RNAs cleaved by RNase L and ribonuclease A

Recombinant human RNase L expressed in insect cells and trimer 2-5A [p_3_5’A(2’p5’A)_2_] were purified as previously described (30,45,46). Bovine pancreatic RNase A was obtained from Ambion. Viral RNAs (100 nM) were incubated at 30°C in reactions (50 µl of volume) containing cleavage buffer (25 mM Tris–HCl [pH 7.4], 100 mM KCl, 10 mM MgCl_2_, 40 µM ATP, 7 mM βME), RNase A (0.5 nM), or RNase L (25 nM), and 2-5A (25 nM). These reaction conditions were optimized empirically to give limited and increasing amounts of viral RNA cleavage over time. RNase A, even at relatively low concentrations, cleaved the viral RNAs readily (0.5 nM RNase A/100 nM viral RNA). Higher concentrations of RNase L (25 nM) were needed for optimal enzymatic activity, due in part to the required dimerization of RNase L following 2-5A binding (47). Increased amounts of RNase L (50 nM) and 2-5A (50 nM) were used in reactions containing PV RNA to overcome the competitive inhibitor of RNase L (30,32). Reactions were terminated after the indicated periods of time (0, 2.5, 5, 10 and 20 min) with the addition of 150 µl of 0.5% sodium dodecyl sulphate (SDS) buffer (0.5% SDS, 10 mM Tris–HCl [pH 7.5], 1.25 mM EDTA [pH 8], 100 mM NaCl). After phenol:chloroform:isoamyl alcohol (P:C:I) extraction and ethanol precipitation, 2.5 µg of RNA from each reaction was fractioned by electrophoresis in 1.2% agarose (MOPS-formaldehyde). Ethidium bromide and UV light revealed the location of RNAs in the gel.

### RNA from uninfected and PV-infected W12 and M25 HeLa cells

HeLa cells transfected with pcDNA3 vectors expressing wild-type RNase L (W12 HeLa cells) or a dominant-negative R667A mutant form of RNase L (M25 HeLa cells) were grown as previously described (30) in Dulbecco’s Modified Eagle’s medium (DMEM; Gibco) containing 10% fetal bovine serum, 250 µg per ml G418, 100 U per ml penicillin and 100 µg per ml streptomycin (Hyclone).

Cells were infected with 10 plaque forming units (PFU) per cell of PV diluted in phosphate buffered saline (∼1.2 × 10^6^ cells per 35 mm well). Following 1 h of virus adsorption at room temperature, the inoculum was removed and replaced with 2 ml of DMEM containing fetal bovine serum, pen-strep and G418. Mock infections were performed using phosphate buffered saline without virus. Mock-infected and PV-infected cells were incubated at 37°C. At the indicated times (0, 2, 4, 6 and 8 h after adsorption), RNA was isolated from the cells using guanidine thiocyanate disruption (4 M guanidine thiocyanate, 25 mM sodium citrate, 0.5% N-laurylsarcosine and 0.1 M βME), P:C:I extraction and ethanol precipitation. Host and viral RNAs in each sample were separated by agarose gel electrophoresis and visualized using ethidium bromide and UV light. PV was isolated from a parallel set of cells and quantified by plaque assays to monitor the magnitude and kinetics of virus replication (30).

### 2′, 3′-cyclic phosphate cDNA synthesis and Illumina sequencing

*Arabidopsis thaliana* tRNA ligase and yeast 2′-phosphotransferase (Tpt1) were purified as previously described (41). An RNA linker with an 8-base unique molecular identifier (UMI) (5′-PO4-NNNNNNNN-AGA UCG GAA GAG CGU CGU GUA GGG AAA GAG-Amino Modification-3′ [NNNNNNNN=UMI] [Integrated DNA Technologies]) was attached to host and viral RNAs in reactions (20 µl of volume) containing buffer (50 mM Tris–HCl [pH 7.5], 40 mM NaCl, 5 mM MgCl_2_, 0.03 mM ATP and 1 mM DTT), *A. thaliana* tRNA ligase (0.75 µM), Riboguard (Epicenter) at 1U per µl and RNA from *in vitro* reactions (2 µg of viral RNA fragments) or RNA from cells (5–8 µg of total cellular RNA). Saturating RNA linker concentrations (20 µM), in excess to RNA fragments with 2′, 3′-cyclic phosphates, maximized ligation efficiencies (data not shown). After incubation at 37°C for 1 h, RNAs were purified using P:C:I extraction and ethanol precipitation. GlycoBlue (Ambion) was included in all ethanol precipitation steps associated with the preparation of cDNA libraries. The RNAs were fragmented for 10 min at 70°C using 1X Fragmentation Reagents (Ambion), followed by an ethanol precipitation. Because a 2′-phosphate persists at the junction between RNA fragments and RNA linkers, fragmented RNAs were dephosphorylated at 30°C for 1 h in reactions (20 µl of volume) containing Tpt1 buffer (20 mM Tris–HCl [pH 7.5], 5 mM MgCl_2_, 0.1 mM DTT and 0.4% Triton X-100), NAD+ (10 mM) and Tpt1 (4.5 µM). These RNAs were P:C:I extracted and ethanol precipitated.

Following the ligation and Tpt1 reactions, RNAs were resuspended in denaturing sample buffer (95% formamide, 18 mM EDTA, 0.025% SDS and 0.025% each bromophenol blue and xylene cyanol), heat denatured (10 min at 65°C) and fractioned on a 6% urea-polyacrylamide gel. RNAs in the gel were visualized using SYBR-Gold (Invitrogen) and blue light transillumination. RNAs 100–500 nt in length were excised from the gel, crushed into a slurry with 0.3 M sodium acetate and incubated at 42°C for 2 h. RNA was purified from polyacrylamide by running samples through DTR cartridges (EdgeBio) followed by ethanol precipitation.

Superscript III (Invitrogen) and a DNA primer (5′-AAT GAT ACG GCG ACC ACC GAG ATC TAC ACT CTT TCC CTA CAC GAC GCT C-3′) were incubated with the gel-purified RNAs to make cDNA (per the manufacturer’s instructions). Exonuclease I (0.5 U) and recombinant shrimp alkaline phosphatase (0.25 U) (USB/Affymetrix) were added to each RT reaction, followed by additional incubation at 37°C for 30 min, to remove excess DNA primer and dNTPs. RT reactions were then heated to 95°C to inactivate the enzymes. RNAs were hydrolyzed using 1 N NaOH treatment at 100°C for 10 min and neutralized with 1 N HCl. cDNA was P:C:I extracted and ethanol precipitated.

A DNA linker was attached to the 3′-end of the cDNA in reactions (10 µl of volume) containing 1 µM miRNA cloning linker 1 (5′-rApp-CTG TAG GCA CCA TCA AT-ddC-3′ [Integrated DNA Technologies]), 10 units of T4 RNA ligase I (New England Biolabs) and T4 RNA ligase buffer (50 mM Tris-HCl [pH 7.5], 10 mM MgCl_2_ and 1 mM DTT). Linked cDNAs were P:C:I extracted and ethanol precipitated. Linked cDNAs were resuspended in formamide sample buffer and fractioned on a 6% urea-polyacrylamide gel. cDNAs >150 bases long were gel purified and resuspended in 30 µl of water.

Polymerase chain reaction (PCR) was used to amplify cDNA libraries before Illumina sequencing. PCR reactions (50 µl) contained 1 U Phusion High Fidelity DNA polymerase (New England Biolabs), HF or GC Phusion buffer, 0.4 mM dNTPs, 5–8 µl of cDNA template, 0.8 µM forward indexed primer (5′-CAA GCA GAA GAC GGC ATA CGA GAT XXX XXX GTG ACT GGA GTT CAT TGA TGG TGC CTA CAG-3′ where XXX XXX = a distinct 6-base index sequence used to distinguish one cDNA library from another when mixing multiple libraries into one Illumina sequencing run), 0.8 µM reverse primer (5′-AAT GAT ACG GCG ACC ACC GAG ATC TAC ACT CTT TCC CTA CAC GAC GCT CTT CCG ATC T-3′) and 1.0–4.6% DMSO. Thermal cycling conditions were an initial 98°C for 30 s, 28–30 cycles of [98°C for 5 s, 59°C for 20 s and 72°C for 30 s], a final extension at 72°C for 10 min and a 4°C hold.

PCR products 250–1000 bp in length were gel purified from a 6% nondenaturing polyacrylamide gel and resuspended in 10 mM Tris–HCl [pH 8.5] with 0.1% Tween-20. The amounts of DNA in each library were determined using the Qubit fluorometer (Invitrogen). Five to seven DNA libraries were combined for multiplexed sequencing for a combined total of 10 nM of DNA for each Illumina sequencing run. A multiplexing index read primer (5′-CTG TAG GCA CCA TCA ATG AAC TCC AGT CAC-3′) was used to identify the library after sequencing on the MiSeq or GAIIx (Illumina, Inc).

### Bioinformatic analyses

Illumina sequence data were trimmed of residual linker sequences and then aligned to the following sequences using Bowtie (48) with a 5′-flag (−5 8) to account for the 8-base UMI, and the -m 1 –best –strata options: PV type 1 Mahoney (V01149.1), hepatitis C genotype 1a (AF009606.1), human genome build 36.1, 28S rRNA (NR_003287.2), 18S rRNA (NR_003286.2), 5.8S rRNA (NR_003285.2), 5S rRNA (NR_023371.1) and U6 snRNA (NR_046491.1). To determine the original number of RNA molecules in the library, the random 8-base UMI at the 5′-end of each aligned sequence was counted (49). cDNA sequences with distinct UMIs were counted, while cDNA sequences with repetitive UMIs (same UMI sequence seen more than once on any particular RNA fragment) were discarded from further analyses. UMI-corrected cDNA analyses eliminate PCR bias (where one cDNA molecule is amplified and sequenced multiple times) (49).

To determine the position and frequency of endonuclease cleavage sites in host and viral RNAs, the 3′-end of each cDNA read was plotted against the nucleotide position of each genome using R (50). For RNase L-cleaved HCV, and RNase A-cleaved HCV and PV RNA data, the signal from the ‘no 2-5A’ and ‘no RNase A’ was subtracted from the 0, 2.5, 5, 10 and 20 min data sets. The 3′-dinucleotides of aligned reads were quantified using the UMIs and the sum of each of the 16 possible dinucleotides was divided by the total number of UMI-corrected reads for each RNA of interest and multiplied by 100 to get a percentage.

### NCBI GEO

The data discussed in this publication have been deposited in NCBI's Gene Expression Omnibus (Cooper *et al.*, 2014) and are accessible through GEO Series accession number GSE52489 (51).

## RESULTS

### 2′, 3′-cyclic phosphate cDNA synthesis and Illumina sequencing methods

RNase L, RNase A and other metal-ion–independent endoribonucleases target single-stranded regions of RNA, leaving 2′, 3′-cyclic phosphates at the end of RNA fragments (Table 1). We exploited the 2′, 3′-cyclic phosphates at RNA cleavage sites to make cDNA libraries suitable for Illumina sequencing (Supplementary Figure S1).

### Viral RNAs cleaved with purified RNase L and RNase A

**Figure 1.**
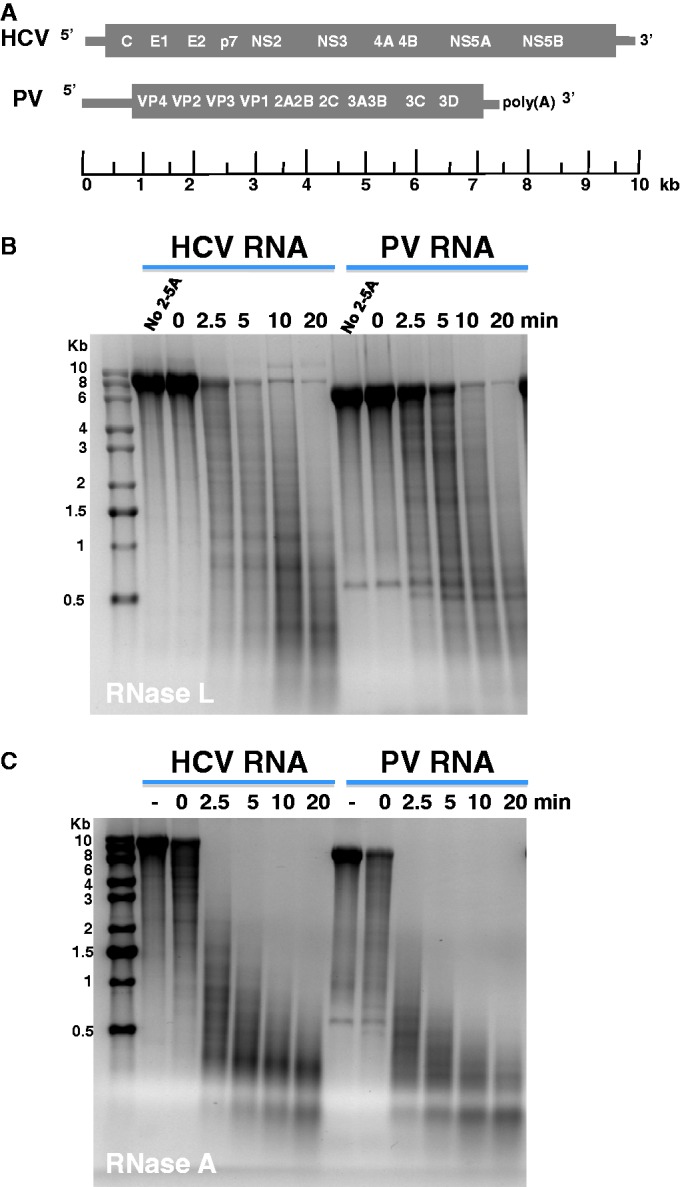
Viral RNA fragments produced by RNase L and RNase A. HCV and PV RNAs were incubated with RNase L and RNase A to produce RNA fragments for 2′, 3′-cyclic phosphate cDNA synthesis and sequencing. Agarose gel electrophoresis and ethidium bromide staining revealed the size of viral RNA fragments. (**A**) Diagram of HCV and PV RNAs. HCV RNA is 9648 bases long. PV RNA is 7500 bases long. (**B**) Viral RNAs incubated with RNase L. HCV and PV RNAs were incubated with RNase L for 20 min in the absence of 2-5A (no 2-5A), or with RNase L and 2-5A for 0, 2.5, 5, 10 and 20 min. (**C**) Viral RNAs incubated with RNase A. HCV and PV RNAs were incubated for 20 min in the absence of RNase A (−), and the presence of RNase A for 0, 2.5, 5, 10 and 20 min.

Initially, we used viral RNAs and purified endoribonucleases to optimize and validate the 2′, 3′-cyclic phosphate cDNA synthesis and Illumina sequencing methods. HCV and PV RNAs were incubated for 0–20 min in reactions containing RNase L and 2-5A or RNase A, followed by agarose gel electrophoresis to characterize the RNA fragments (Figure 1). RNase L and RNase A generated viral RNA fragments ranging from 100 to several thousand bases in length. Notably, HCV and PV RNA fragments with distinct sizes were evident, consistent with nonrandom cleavage of the viral RNAs (Figure 1).

### Frequency, location and dinucleotide specificity of endoribonuclease cleavage sites in viral RNAs

The viral RNAs from each time point shown in Figure 1 were analyzed by 2′, 3′-cyclic phosphate cDNA synthesis and Illumina sequencing (Supplemantary Tables S1 and S2 and Supplementary Figures S2–S5). RNase L and RNase A cleavage sites in the viral RNAs were extremely reproducible across RNA samples from independent time points (Supplementary Figures S2 and S3).

**Figure 2.**
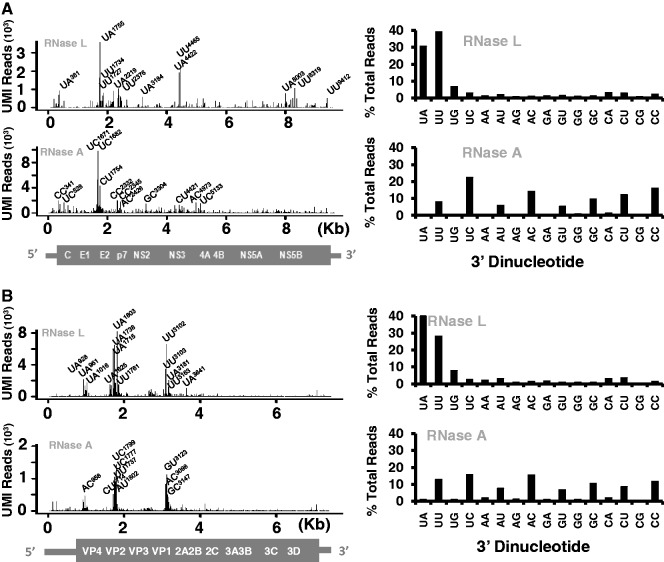
Frequency, location and dinucleotide specificity of cleavage sites in viral RNAs. 2′, 3′-cyclic phosphate cDNA synthesis and Illumina sequencing was used to analyze the viral RNA fragments shown in Figure 1. (**A**) Frequency, location and dinucleotide specificity of endoribonuclease cleavage sites in HCV RNA (from 20 min samples). (**B**) Frequency, location and dinucleotide specificity of cleavage sites in PV RNA (from 20 min samples).

RNase L cleaved HCV and PV RNAs predominantly at UpN dinucleotides (UA and UU > UG), with prominent amounts of cleavage at distinct locations in the viral RNAs (Figure 2). Pyrimidines were the most common nucleotides at the end of viral RNA fragments produced by RNase A (Figure 2), consistent with the known specificity of RNase A (52). RNase L and RNase A targeted single-stranded regions of viral RNA (Supplementary Figures S4 and S5). A peak of cleavage was evident in the E2 gene of HCV RNA, with especially large amounts of RNase L cleavage at UA^1755^, UA^4422^ and UU^4465^ and especially large amounts of RNase A cleavage at UC^1671^, UC^1682^ and CU^1754^ (Figure 2A). Three independent peaks of cleavage were evident in PV RNA; region 1 (884–1042 nt), region 2 (1705–1827 nt) and region 3 (3088–3221 nt) (Figure 2B and Supplementary Figure S5). As highlighted in Supplementary Figure S5, UA^961^ was the most frequent RNase L cleavage site within region 1 (1108 UMI reads after 5 min of cleavage), UA^1803^ was the most frequent RNase L cleavage site within region 2 (4223 UMI reads after 5 min of cleavage) and UU^3102^ was the most frequent RNase L cleavage site within region 3 (5347 UMI reads after 5 min of cleavage). Intriguingly, these regions of PV RNA encode portions of the capsid proteins recognized by neutralizing antibodies (Supplementary Figure S6).

Consistent with increasing amounts of viral RNA cleavage over time, the number of UMI-corrected cDNA reads at prominent cleavage sites in HCV and PV RNAs increased coordinate with the time of incubation with RNase L (Supplementary Figures S2 and S4 for HCV RNA/Supplementary Figures S3 and S5 for PV RNA). This is most clearly presented in Supplementary Figure S4, focusing on four prominent RNase L cleavage sites in HCV RNA. The number of UMI-corrected cDNA reads at UA^1755^, UA^4422^, UU^4465^ and UU^4466^ increased coordinate with the time of incubation with RNase L (Supplementary Figure S4). Note that UU^4465^ and UU^4466^ are adjacent cleavage sites, where cleavage at UU^4465^ would preclude detectable cDNA reads at UU^4466^. These data suggest that viral RNAs were partially digested, even after 20 min of incubation with RNase L, and that prominent cleavage sites detected at early times did not disappear after more prolonged periods of incubation.

### RNA from uninfected and PV-infected HeLa cells

**Figure 3.**
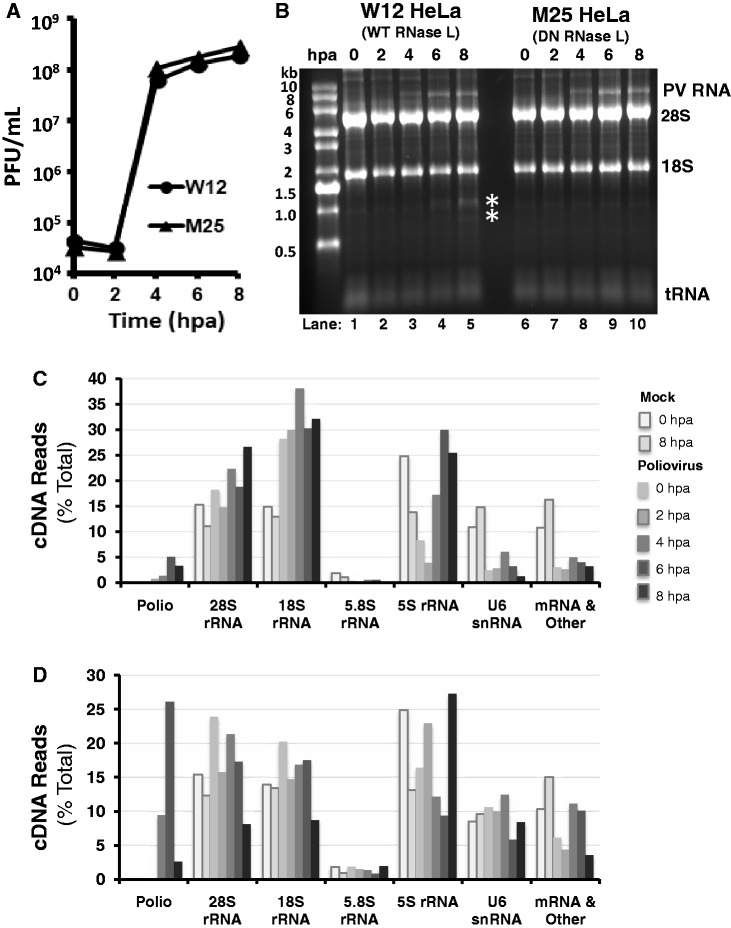
Host and viral RNA from mock-infected and PV-infected HeLa cells. RNA was isolated from mock-infected and PV-infected HeLa cells for 2′, 3′-cyclic phosphate cDNA synthesis and Illumina sequencing. (**A**) PV infection. W12 and M25 HeLa cells were infected with PV using 10 PFUs per cell. PV titers determined by plaque assay and plotted versus time (hpa). (**B**) RNA from PV-infected HeLa cells. RNA was isolated from infected cells, fractionated by agarose gel electrophoresis and visualized using ethidium bromide and UV light. (**C** and **D**) cDNA reads from W12 (C) and M25 (D) HeLa cells. The RNAs shown in Figure 3B were used for 2′, 3′-cyclic phosphate cDNA synthesis and Illumina sequencing. Amounts of host and viral cDNA in each sample are plotted (data from Supplementary Table S3).

After optimizing and validating the 2′, 3′-cyclic phosphate cDNA synthesis and Illumina sequencing methods using purified endoribonucleases, we used the methods to characterize RNA from uninfected and PV-infected HeLa cells (Figure 3). W12 HeLa cells express wild-type RNase L, whereas M25 HeLa cells express a dominant-negative mutant form of RNase L (R667A mutation). We previously established that RNase L activity, which requires 2-5A from dsRNA-activated oligoadenylate synthetase, is provoked during the course of PV infection in W12 HeLa cells, whereas RNase L activity remains undetectable during the course of PV infection in M25 HeLa cells (30). The kinetics and magnitudes of PV replication in W12 and M25 HeLa cells are similar (Figure 3A). When RNAs from the PV-infected cells were analyzed by agarose gel electrophoresis, the accumulation of viral RNA was evident at 4–8 h post adsorption (hpa) (Figure 3B). rRNA fragments characteristic of RNase L activity were evident in RNAs from PV-infected W12 HeLa cells at 6 and 8 hpa, but these rRNA fragments were not detected in PV-infected M25 HeLa cells (Figure 3B, asterisks indicate the location of rRNA fragments characteristic of RNase L activity).

cDNA libraries were prepared and sequenced using the RNAs from HeLa cells (Figure 3C and D and Supplementary Table S3). cDNA reads corresponding to PV RNA increased from undetectable levels at early times after infection to 5% of the cDNA at 6 hpa in W12 HeLa cells and 26.1% of the cDNA at 6 hpa in M25 HeLa cells (Figure 3C and D and Supplementary Table S3). Abundant amounts of cDNA in each sample aligned to rRNAs (28S/18S/5.8S/5S rRNAs) and U6 snRNA (Figure 3C and D and Supplemantary Table S3). cDNA reads corresponding to host mRNAs and other nonribosomal RNAs were present in each sample, ranging from 2.6% of all cDNA from PV-infected W12 HeLa cells at 2 hpa to 16.3% of cDNA in mock-infected W12 HeLa cells at 8 hpa.

### Endoribonuclease cleavage sites in PV RNAs isolated from HeLa cells

**Figure 4.**
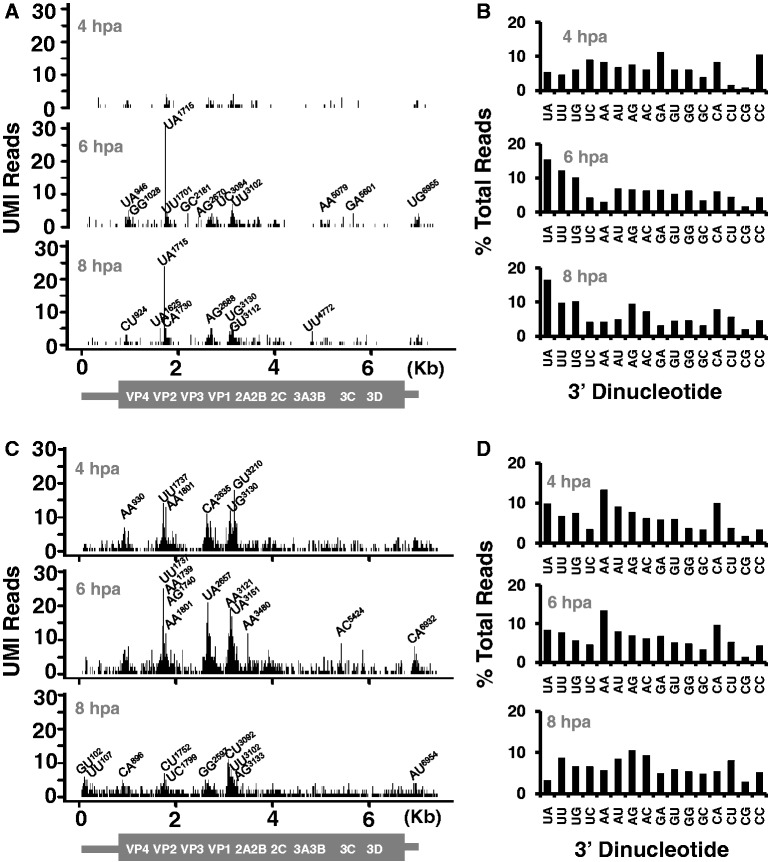
Endoribonuclease cleavage sites in PV RNA isolated from HeLa cells. RNAs from PV-infected HeLa cells (Figure 3B) were used for 2′, 3′-cyclic phosphate cDNA synthesis and Illumina sequencing. (**A**) Location and frequency of cleavage sites in PV RNA from W12 HeLa cells. X-axis: Nucleotide position in PV RNA. Y-axis: Number of distinct UMI-tagged linkers detected at each cleavage site. (**B**) Dinucleotide specificity of cleavage sites in PV RNA from W12 HeLa cells. X-axis: Dinucleotide at the 3′-end of PV RNA fragments (adjacent to 8 base UMI sequence in RNA linkers as illustrated in Supplementary Figure S1). Y-axis: Percent of PV cDNA reads. (**C**) Location and frequency of cleavage sites in PV RNA from M25 HeLa cells. X-axis: Nucleotide position in PV RNA. Y-axis: Number of distinct UMI-tagged linkers detected at each cleavage site. (**D**) Dinucleotide specificity of cleavage sites in PV RNA from M25 HeLa cells. X-axis: Dinucleotide at the 3′-end of PV RNA fragments. Y-axis: Percent of PV cDNA reads.

Discrete regions of PV RNA were targeted by endoribonucleases both *in vitro* (Figure 2B and Supplementary Figure S3) and *in vivo* (Figure 4). One prominent RNase L-dependent cleavage site at UA^1715^ was evident in PV RNA isolated at 6 and 8 hpa from W12 HeLa cells (Figure 4A). A second potential RNase L-dependent cleavage site at UU^3102^ was evident in PV RNA isolated at 6 and 8 hpa from W12 HeLa cells (Figure 4A); however, there was substantial RNase L-independent cleavage at UU^3102^ in M25 HeLa cells (Figure 4C), making it uncertain as to the enzyme(s) responsible for cleavage at this site in cells. UA^1715^ and UU^3102^ are prominent RNase L cleavage sites identified using purified RNase L (Figure 2B and Supplementary Figure S5). Surprisingly, there were no other obvious RNase L-dependent cleavage sites in PV RNA isolated from W12 HeLa cells. The increased frequency of UA and UU dinucleotides in cDNAs from W12 HeLa cells at 6 and 8 hpa was consistent with the activation of RNase L at these times (compare Figure 4B and D). RNase L-independent cleavage sites in PV RNA from HeLa cells mapped to the same regions of PV RNA as the cleavage sites found using purified RNase L and RNase A, with notable peaks of cleavage in the 5′-half of the open-reading frame (compare Figure 2B with Figure 4A and C). Potential reasons for these hypersensitive regions in PV RNA are considered in the discussion. Remarkably, PV cDNA reads from HeLa cells corresponded to cleavage sites exclusively within positive-strand RNA; no cDNA reads were found corresponding to cleavage sites within PV negative-strand RNA.

### Endoribonuclease cleavage sites in rRNAs isolated from HeLa cells

**Figure 5.**
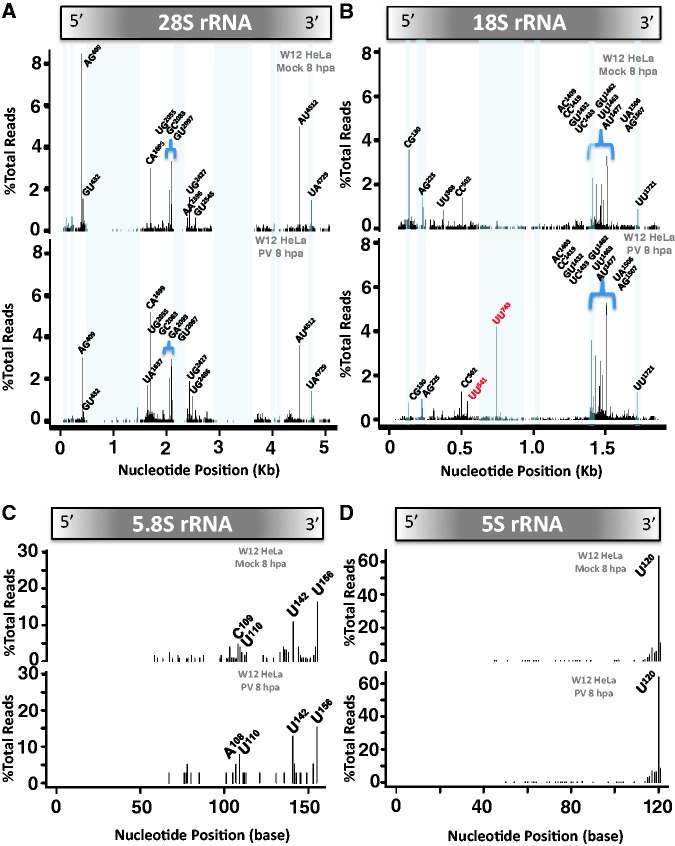
Endoribonuclease cleavage sites in rRNAs from W12 HeLa cells. RNAs from mock-infected and PV-infected W12 HeLa cells (Figure 3B) were used for 2′, 3′-cyclic phosphate cDNA synthesis and Illumina sequencing. The location and frequency of cleavage sites in 28S rRNA (**A**), 18S rRNA (**B**), 5.8S rRNA (**C**) and 5S rRNA (**D**) are shown for mock-infected and PV-infected RNA samples isolated at 8 hpa. X-axis: Nucleotide position of each RNA. Y-axis: Percentage of total UMIs at each cleavage site. Dinucleotides at the 3′-end of abundant RNA fragments are annotated at the corresponding positions in the graphs. The locations of GC-rich expansion segments are highlighted by light blue rectangles. RNase L cleavage sites are highlighted in red.

**Figure 6.**
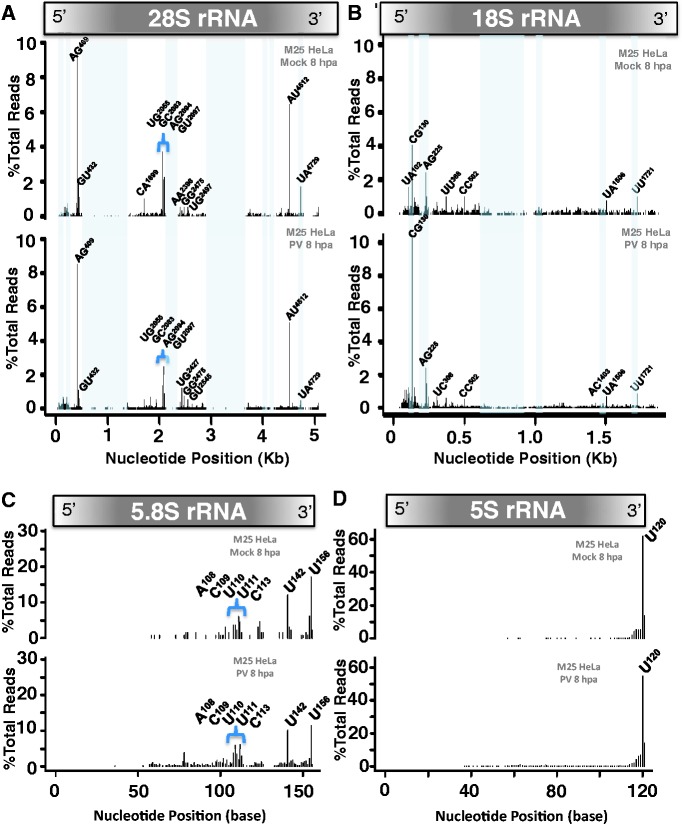
Endoribonuclease cleavage sites in rRNAs from M25 HeLa cells. RNAs from mock-infected and PV-infected M25 HeLa cells (Figure 3B) were used for 2′, 3′-cyclic phosphate cDNA synthesis and Illumina sequencing. The location and frequency of cleavage sites in 28S rRNA (**A**), 18S rRNA (**B**), 5.8S rRNA (**C**) and 5S rRNA (**D**) are shown for mock-infected and PV-infected RNA samples isolated at 8 hpa. X-axis: Nucleotide position of each RNA. Y-axis: Percentage of total UMIs at each cleavage site. Dinucleotides at the 3′-end of abundant RNA fragments are annotated at the corresponding positions in the graphs. The locations of GC-rich expansion segments are highlighted by light blue rectangles.

The location and frequency of endoribonuclease cleavage sites in rRNAs was largely consistent across all of the RNA samples from uninfected and PV-infected HeLa cells (Figures 5 and 6 and Supplementary Figures S7–S12). 28S rRNA was cleaved in an RNase L-independent fashion at specific sites, most frequently at AG^409^, GU^432^, CA^1699^, UG^2055^, GC^2083^, GA^2093^, GU^2097^, AA^2396^, UG^2427^, AU^4512^ and UA^4729^ (Figures 5A and 6A; Supplementary Figures S7 and S8 and Supplementary Table S4). There were no obvious RNase L-dependent cleavage sites in 28S rRNA under the conditions of this experiment. Interestingly, expansion segments of 28S rRNA were largely resistant to cleavage (Figures 5A and 6A, location of expansion segments highlighted in blue).

18S rRNA was cleaved reproducibly at specific sites, including two RNase L-dependent cleavage sites (Figures 5B and 6B; Supplementary Figures S9 and S10 and Supplemantary Table S5). The most common cleavage sites identified in 18S rRNA include CG^130^, AG^225^, CC^502^, **UU^541^**, **UU^743^**, AC^1403^, CC^1419^, GU^1432^, UC^1433^, GU^1462^, UU^1463^, AU^1477^, UA^1506^, AG^1507^ and UU^1721^ (Figures 5B and 6B and Supplementary Table S5). UU^541^ and UU^743^ were clearly RNase L-dependent cleavage sites based on their increased frequency in PV-infected W12 HeLa cells at 6 and 8 hpa and the low frequency of cleavage at these sites in RNA isolated from uninfected and PV-infected M25 HeLa cells (Supplementary Table S5).

Reproducible distribution of RNase L-independent cleavage sites was exhibited by 5.8S rRNA (Figures 5C and 6C; Supplementary Figure S11 and Supplementary Table S6), which had relatively few 2′, 3′-cyclic phosphates relative to other rRNAs (Figure 3C and D and Supplementary Table S3). The most common cleavage sites identified in 5.8S rRNA include cleavage at A^80^, A^108^, C^109^, U^110^, U^111^, C^113^, A^137^, U^142^ and U^156^ (Figures 5C and 6C and Supplementary Table S6). Notably, 2′, 3′-cyclic phosphates were frequently detected at the 3′-end of mature 5.8S rRNA (Figures 5C and 6C and Supplementary Figure S11).

A striking distribution of RNase L-independent cleavage sites, with the majority of signal at the 3′-end of mature 5S rRNA (Figures 5D and 6D and Supplementary Figure S12), was exhibited by 5S rRNA, which consistently had large amounts of 2′, 3′-cyclic phosphate-dependent cDNA relative to other RNAs from HeLa cells (Figure 3C and D and Supplementary Table S3).

**Figure 7.**
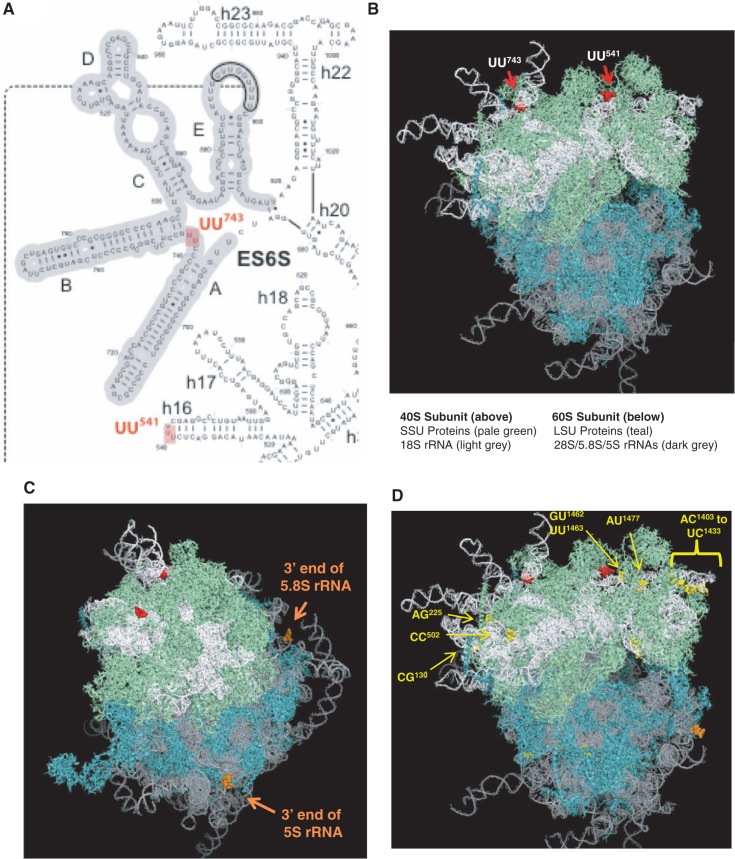
Cleavage sites mapped onto rRNA secondary and tertiary structures. Secondary and tertiary structures from Anger *et al.* (42). (**A**) RNase L cleavage sites in 18S rRNA secondary structure. Portion of 18S rRNA structure highlighting the location of RNase L cleavage sites. (**B**) Location of RNase L cleavage sites in 80S ribosome tertiary structure. RNase L-dependent cleavage sites highlighted in red spheres. (**C**) 3′-end of 5.8S and 5S rRNAs. 3′-end of 5.8S and 5S rRNAs highlighted in orange spheres. (**D**) RNase L-independent cleavage sites. Some representative RNase L-independent cleavage sites highlighted in yellow spheres.

The location of these RNase L-dependent and RNase L-independent endoribonuclease cleavage sites were mapped onto the secondary structures of rRNAs (Supplementary Figures S13 and S14), as well as the tertiary structure of 80S ribosomes (Figure 7) (27). RNase L-dependent cleavage sites within 18S rRNA map to single-stranded regions of RNA on the surface of the 40S subunit (Figure 7A and B). Likewise, the 3′-ends of 5.8S and 5S rRNAs are located on the surface of the 60S subunit (Figure 7C). RNase L-independent cleavage sites mapped to various locations on the surface of 40S subunits (Figure 7D). Cleavage sites were less frequently present at the surface of 60S subunits (Figure 7D).

### U6 snRNA

U6 snRNA accounts for a substantial amount of cDNA in each sample from HeLa cells, ranging from 8.5 to 14.8% of total cDNA reads in mock-infected HeLa cells (Figure 3C and D and Supplementary Table S3). The U6 snRNA cDNA reads in our libraries align almost exclusively to the 3′-end of mature U6 snRNA (Supplementary Figure S15). U6 snRNA, an RNA polymerase III (Pol III) transcript, is posttranscriptionally modified by a nuclease encoded by gene C16orf57, which leaves 2′, 3′-cyclic phosphates at the end of mature U6 snRNAs (6,7). The abundant amounts of cDNA in our libraries corresponding to this particular modification of U6 snRNA further validate the reliability of the 2′, 3′-cyclic phosphate cDNA synthesis and Illumina sequencing methods.

## DISCUSSION

RNase L, like other metal-ion–independent endonucleases, is expected to produce RNA fragments with 2′, 3′-cyclic phosphate termini (Table 1). Our data confirm that RNase L, like RNase A, produces RNA fragments with 2′, 3′-cyclic phosphate termini. We used 2′, 3′-cyclic phosphate cDNA synthesis and Illumina sequencing methods to identify and quantify metal-ion–independent endoribonuclease cleavage sites within host and viral RNAs. We optimized and validated the methods using viral RNAs cleaved with purified RNase L, viral RNAs cleaved with purified RNase A and RNA from uninfected and PV-infected HeLa cells (Figures 1–7 and Supplementary Tables S1–S6). Overall, these methods reliably and reproducibly reveal the location and relative frequency of 2′, 3′-cyclic phosphates in host and viral RNAs. These cDNA synthesis and Illumina sequencing methods did not detect RNA fragments corresponding to RNase P or RNase MRP activity, consistent with the specificity of the method for RNA fragments with 2′, 3′-cyclic phosphates.

Several lines of evidence suggest that cyclic phosphates within host and viral RNAs were due to metal-ion–independent endonucleolytic cleavage of RNA within cells rather than cleavage during RNA processing or within linker reactions. Cyclic phosphates were not frequent in viral RNA transcripts isolated from control reactions lacking endonucleases, confirming the absence of endonuclease activity within RNA linker reactions or other steps of RNA processing. The integrity of host and viral RNAs purified from cells was confirmed by agarose gel electrophoresis (Figure 3B), host and viral RNAs exhibited cell-type–specific cleavage sites consistent with RNase L activity in virus-infected W12 HeLa cells, as compared with virus-infected M25 HeLa cells, which express a dominant-negative form of RNase L (Figures 3B, 4–6/RNase L-dependent cleavage of PV RNA and 18S rRNA in W12 HeLa cells), and cyclic phosphates at the end of U6 snRNA are consistent with the enzymatic activity of C16orf57 (Supplementary Figure S15), a cellular enzyme known to leave cyclic phosphates at the end of mature U6 snRNA in cells (6,7). Nonetheless, it is important to note that divalent metal ions can promote RNA cleavage at specific sites, leaving cyclic phosphates (53). Thus, in addition to metal-ion–independent endoribonucleases, some of the RNA cleavage sites revealed by 2′, 3′-cyclic phosphate cDNA synthesis and Illumina sequencing could be due to metal RNA binding pockets within RNAs. In fact, Pb^2+^-dependent cleavage of 28S rRNA at AG^409^ has been reported (54). This is a prominent and reproducible cleavage site within our data sets (Figures 5A and 6A and Supplementary Figures S7A and S8A). Thus, a metal binding pocket at this site in 28S rRNA, under physiologic conditions within cells, may lead to rRNA cleavage. Carefully controlled experimental variables are needed to attribute specific cleavage sites to particular endonucleases or to metal binding pockets.

### Endoribonuclease cleavage sites in HCV RNA

Our original interest in 2′, 3′-cyclic phosphate cDNA synthesis and Illumina sequencing methods was driven by previous studies of RNase L and HCV RNA. Primer extension reveals RNase L cleavage sites in HCV RNA *in vitro* (27) but this method suffers from several limitations and is unsuitable for detecting RNase L cleavage sites in host and viral RNAs from tissues. To overcome the limitations of primer extension, we developed and validated 2′, 3′-cyclic phosphate cDNA synthesis and Illumina sequencing methods to detect the RNA fragments produced by RNase L and other metal-ion–independent ribonucleases.

RNase L cleavage sites in HCV RNA are efficiently detected by 2′, 3′-cyclic phosphate cDNA synthesis and Illumina sequencing (Figure 2A). The 8 base UMI sequence in RNA linkers, which corresponds to 65 536 unique molecules, allows for precise quantification of cleavage sites (49). UA^1755^, and a small number of other sites in HCV RNA, were consistently linked to large numbers of distinct RNA linkers (Figure 2A, Y-axis, UMI-corrected cDNA reads), whereas most other regions in HCV RNA were cleaved infrequently. These results are consistent with those obtained using primer extension (27); however, the quantitative data from 2′, 3′-cyclic phosphate cDNA synthesis and Illumina sequencing is more reliable than that obtained using primer extension. Quantitative analyses of RNase L cleavage sites in HCV RNA by primer extension were confounded by multiple factors: the use of multiple primers (30 primers across the HCV RNA genome), variable hybridization efficiencies for each primer and decreased sensitivity of cleavage site detection as the distance increased from the site of hybridization. In contrast, quantitative analyses of RNase L cleavage sites in HCV RNA using 2′, 3′-cyclic phosphate cDNA synthesis and Illumina sequencing are extremely precise. Because HCV RNAs from distinct genotypes have variably reduced frequencies of UA and UU dinucleotides (40), the potential role(s) of RNase L in HCV infections and interferon-based antiviral therapies deserves further examination now that a method has been developed to reliably detect and quantify RNase L cleavage sites in host and viral RNAs.

### Endoribonuclease cleavage sites in PV RNA

Endoribonuclease cleavage sites across PV RNA genomes have not been previously described. The striking distribution of RNase L and RNase A cleavage sites in PV RNA (Figure 2B), with prominent amounts of cleavage in discrete regions of the genome, was not expected. The same discrete regions of PV RNA were targeted by endoribonucleases both *in vitro* (Figure 2B) and *in vivo* (Figure 4). Furthermore, one RNase L-specific cleavage site was reliably detected both *in vitro* and *in vivo* (Figures 2B and 4, UA^1715^ in PV RNA). We entertained two hypotheses as we considered these data. First, PV RNA may assume a secondary and tertiary structure that renders some portions of the genome particularly susceptible to single-strand–specific endoribonucleases while rendering other regions resistant (Supplementary Figure S5). Second, antigenic variation in the capsid proteins, as a consequence of antibody selection, could render portions of the capsid genes more sensitive to cleavage by single-strand–specific endoribonucleases, due to the counter-selective forces of antigenic variation and resistance to endoribonucleases (Supplementary Figure S6). The predicted secondary structures of HCV and PV RNAs do explain much of the data (55), as metal-ion–independent endoribonucleases do not cleave dsRNA portions of viral RNA (Supplementary Figures S4 and S5). In addition, atomic force microscopy of HCV and PV RNA genomes reveals globular structures, where some regions of the viral RNA genomes might be more exposed to endoribonucleases than others (56,57). Intriguingly, antibody neutralization escape mutations are found within and near endonuclease susceptible regions of PV RNA (58,59); however, the peaks of endonucleolytic cleavage in the PV capsid genes do not overlap exclusively with the location of RNA changes associated with antigenic variation (Supplementary Figure S6). A complete understanding of the discrete distribution of endoribonuclease cleavage sites in PV RNA awaits further investigation.

### Endoribonuclease cleavage sites in rRNA

A constellation of RNase L-dependent and RNase L-independent cleavage sites in rRNAs was revealed by 2′, 3′-cyclic phosphate cDNA synthesis and Illumina sequencing (Figures 5 and 6; Supplementary Figures S7–S14 and Supplementary Tables S4–S6). Unlike many studies, which actively exclude rRNAs from deep sequencing experiments to enrich for other RNA populations, we intentionally retained rRNAs in our study to identify RNase L-dependent and RNase L-independent cleavage sites in rRNAs. Although we did not detect RNase L-dependent cleavage sites in 28S rRNA under the conditions of our experiment, others have reported RNase L-dependent cleavage of 28S rRNA (60,61). We did identify two RNase L-dependent cleavage sites in 18S rRNA, UU^541^ and UU^743^ (Figures 5B and 7A and B). Cleavage of 18S rRNA (1869 bases long) at these sites would produce rRNA fragments of 541, 743, 1126 and 1328 bases long, consistent with the characteristic rRNA fragments observed by agarose gel electrophoresis in this report (Figure 3B), and in previous studies by our lab and others (30,37). As expected for RNase L, the 18S rRNA cleavage sites, UU^541^ and UU^743^, are associated with single-stranded regions of 18S rRNA, on the surface of 80S ribosomes (Figure 7).

A number of RNase L-independent cleavage sites were identified consistently in rRNAs (Figures 5–7; Supplementary Figures S7–S14 and Supplementary Tables S4–S6). Each of the sites mapped to single-stranded regions of rRNA (Supplementary Figures S13 and S14), and most were located at the surface of 40S and 60S ribosomal subunits (Figure 7D). The RNase L-independent cleavage sites of 28S rRNA occurred in core segments of the 28S rRNA, while little cleavage occurred in the GC-rich expansion segments (Figures 5A and 6A), despite the location of expansion segments on the surface of ribosomes (Figure 7). The paucity of cDNA reads in 28S rRNA expansion segments is likely due to the dsRNA-rich characteristics of expansion segments (42) (Supplementary Figure S13/dsRNA is resistant to metal-ion–independent endoribonucleases) and to the reduced frequency of Illumina cDNA reads in GC-rich regions of RNA (62). The paucity of cDNA reads in rRNA expansion segments evident in our data (Figures 5 and 6) is similar to data from others (63).

The functional impact of rRNA cleavage at particular sites is yet to be determined. rRNA cleavage has been associated with RNase L-dependent (37) and RNase L-independent antiviral responses (38), ER-stress (64), apoptosis (65), oxidative stress (66) and the inhibition of translation during spermatogenesis (63). The RNase L cleavage sites identified in this investigation in 18S rRNA (UU^541^ and UU^743^) are distant from the mRNA decoding channel (Figure 7). It is unclear whether cleavage of 18S rRNA at these sites would affect translation. AG^409^ in 28S rRNA, one of the most prominent RNase L-independent cleavage sites in both uninfected and PV-infected HeLa cells (Figures 5A and 6A; Supplementary Figures S7 and S8 and Supplementary Table S4), is analogous to an oxidative stress-induced rRNA cleavage site identified in yeast (66). Yet again, there are no definitive data to indicate how cleavage of 28S rRNA at this site would affect translation. Nonetheless, because decreased translation is a common aspect of antiviral responses, ER-stress, apoptosis and oxidative stress, it is reasonable to speculate that cleavage of rRNAs at these sites might inhibit the translation activity of ribosomes. It is also important to consider the potential role of endonucleases in rRNA decay (67,68). RNase T2 has been implicated in rRNA decay pathways in plants (69) and vertebrates (70).

The 2′, 3′-cyclic phosphates detected at the ends of 5.8S and 5S rRNAs were unexpected. Cyclic phosphates have been described at the 3′-end of U6, 7SK and MRP RNAs, but not at the 3′-end of 5S rRNA (71). Nonetheless, 5S rRNA was among the most common sequences detected in 2′, 3′-cyclic phosphate cDNA libraries (Figure 3C and D and Supplementary Table S3), and almost all of the 5S rRNA signal was associated with the 3′-end of mature 5S rRNA (Figures 5D and 6D and Supplementary Figure S12). The 3′-ends of 5.8S and 5S rRNAs are on the surface of 60S ribosomal subunits (Figure 7C), consistent with the possibility that ribosomes contain RNAs with these terminal modifications. Alternatively, the mature 3′-end of 5S rRNA, in the context of Mdmx complexes, could be modified with 2′, 3′-cyclic phosphates (72). Rex1p, a 3′ to 5′ exonuclease in the RNase D family, is involved with modifying the 3′-ends of both 5S and 5.8S rRNA in yeast; however, RNase D family members are metal-ion–dependent, and thus it is unlikely that a human homolog of Rex1p is responsible for the 2′, 3′-cyclic phosphate found at the end of these rRNAs (73,74).

While the identity of endonucleases responsible for many of the RNase L-independent cleavage sites in rRNA remains to be determined, the cyclic phosphates found at the ends of 5.8S and 5S rRNA may be consistent with the enzymatic activity of the enzyme associated with U6 snRNA maturation (6,7). The cyclic phosphate at the 3′-end of U6 snRNA facilitates interactions with Lsm proteins (75), which promote U6 snRNP maturation (8). Lsm proteins are also implicated in rRNA processing and maturation (76). Therefore, the cyclic phosphates found at the 3′-ends of 5.8S and 5S rRNAs may contribute to their interaction with Lsm proteins and RNP maturation.

### Endoribonuclease cleavage sites in U6 snRNA

U6 snRNA is a Pol III transcript modified posttranscriptionally by an exonuclease that leaves 2′, 3′-cyclic phosphates at the end of mature U6 snRNAs (6,7). The abundant amounts of cDNA in our libraries corresponding to this particular modification of U6 snRNA further validate the reliability of the 2′, 3′-cyclic phosphate cDNA synthesis and Illumina sequencing methods (Figure 3C and D and Supplementary Figure S15).

### Summary

We used 2′, 3′-cyclic phosphate cDNA synthesis and Illumina sequencing to identify and quantify metal-ion–independent endoribonuclease cleavage sites in host and viral RNAs. With these methods, we identified discrete regions of HCV and PV RNA genomes that were profoundly susceptible to RNase L and other single-strand specific endoribonucleases, RNase L-dependent and RNase L-independent cleavage sites within rRNAs, and 2′, 3′-cyclic phosphates at the ends of 5S rRNA and U6 snRNA. We expect these methods will prove useful in the characterization of other metal-ion–independent endoribonucleases, like the NendoU of arteriviruses and coronaviruses whose RNA substrates remain unknown (17), and the T2 endonucleases that target undefined sites in rRNAs (70).

## SUPPLEMENTARY DATA

Supplementary Data are available at NAR Online.

## FUNDING

Mucosal and Vaccine Research Colorado (MAVRC), Golfers Against Cancer, the University of Colorado Cancer Center, the March of Dimes [Basil O’Connor Starter Scholar Award 5-FY10-478 to J.R.H.]; a Damon Runyon-Rachleff Innovation Award [DRR-17-12 to J.R.H.]; the American Cancer Society [RSG-13-216-01-DMC to J.R.H.]; and the National Institutes of Health (NIH) [CA044059 to R.H.S. and AI042189 to D.J.B.]. Funding for open access charge: NIH.

*Conflict of interest statement*. None declared.

## Supplementary Material

Supplementary Data
