# Supplementary material for: Ribonuclease L and metal-ion–independent endoribonuclease cleavage sites in host and viral RNAs
Source: Nucleic Acids Res. 2014 Feb 5;42(8):5202–16. doi: 10.1093/nar/gku118 (PMC4005677; doi:10.1093/nar/gku118)
Supplement: Supplementary Data [file supp_42_8_5202__index.html]

Ribonuclease L and metal-ion–independent endoribonuclease cleavage sites in host and viral RNAs — Supplementary Data 

# Ribonuclease L and metal-ion–independent endoribonuclease cleavage sites in host and viral RNAs

## Supplementary Data

files

**Files in this Data Supplement:**

- Supplementary Data - pdf file
