# Supplementary material for: Ribonuclease L and metal-ion–independent endoribonuclease cleavage sites in host and viral RNAs
Source: Nucleic Acids Res. 2014 Feb 5;42(8):5202–16. doi: 10.1093/nar/gku118 (PMC4005677; doi:10.1093/nar/gku118)
Supplement: Supplementary Data [file supp_gku118_Supplementary_Data_012714.pdf]

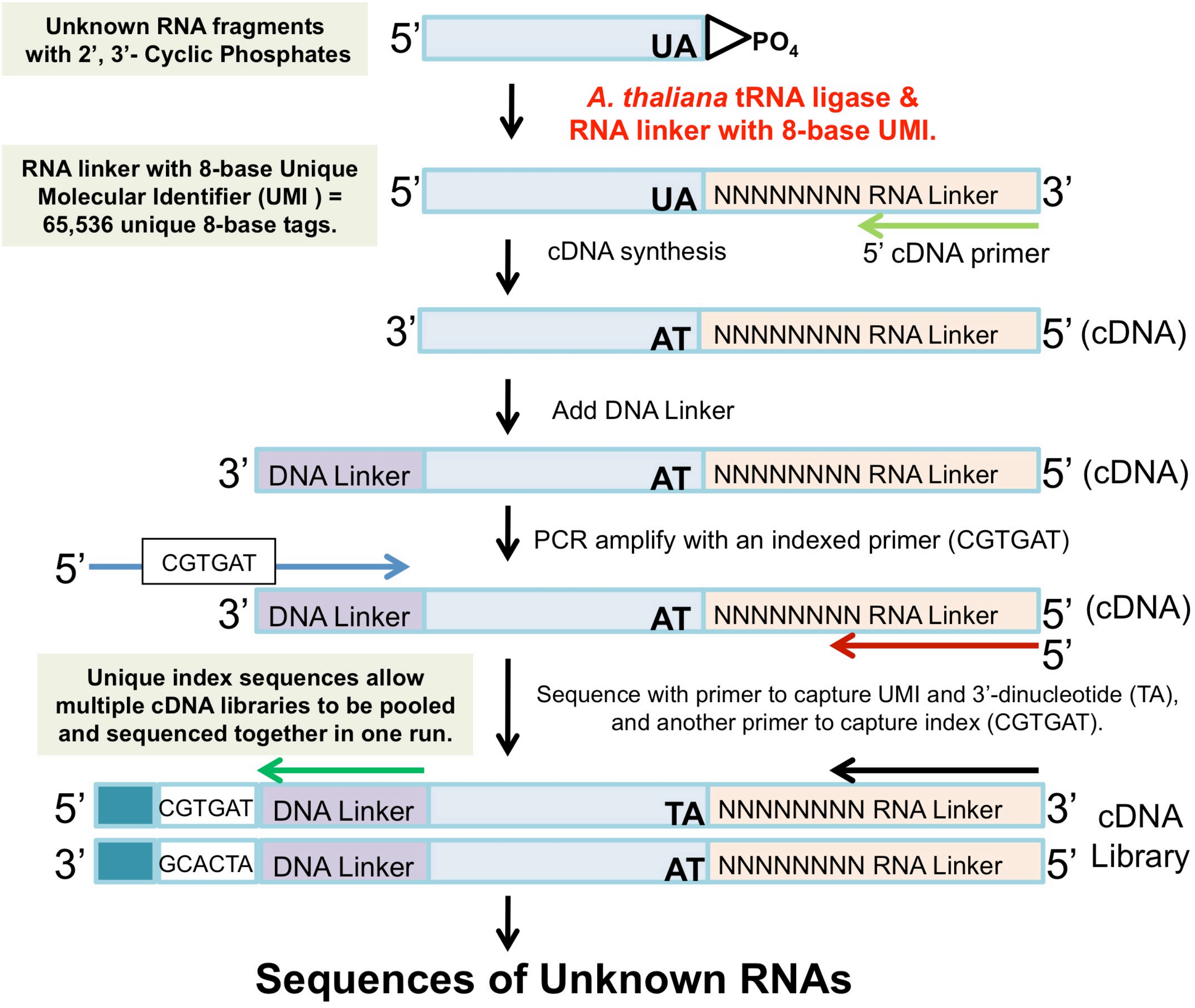

Figure S1. 2', 3'-cyclic phosphate cDNA synthesis and Illumina sequencing methods. Diagram outlining 2', 3'-cyclic phosphate cDNA synthesis and Illumina sequencing methods (1). *Arabidopsis thaliana* tRNA ligase specifically recognizes RNA fragments with 2', 3'-cyclic phosphates. The RNA fragments with 2', 3'-cyclic phosphates are covalently attached to defined RNA linkers containing an 8 base long unique molecular identifier (UMI) sequence (2). After ligation, the 8 base UMI sequence at the 5' end of the RNA linker is juxtaposed next to the endoribonuclease cleavage site at the end of the RNA fragment as illustrated by the UA dinucleotide in the RNA fragment. A DNA primer complementary to the RNA linker is used to make cDNA, followed by the addition of a DNA linker to the 3' end of the cDNA. PCR amplification produces cDNA libraries suitable for Illumina sequencing. Defined index sequences in PCR primers allow for the combination of several cDNA libraries into each Illumina sequencing reaction. As shown in Tables S1 and S2, we typically combine 5, 6 or 7 cDNA libraries into one sequencing reaction. cDNA sequencing, in conjunction with bioinformatic analyses of the data, reveals the frequency and location of endoribonuclease cleavage sites in host and viral RNAs.

Table S1. 2', 3'-Cyclic Phosphate cDNA Synthesis and Illumina Sequencing: HCV RNA* Cleaved by RNase L and RNase A.

RNase L

cDNA Library	Amount** (nM cDNA)	# of Reads (Total)	HCV Specific Reads	UMI-Corrected HCV Reads	Cleavage Sites in HCV RNA
No 2-5A	0.1	27,607	13,786	852	n/a
0 min	0.1	34,145	26,761	1,019	527
2.5 min	0.5	394,301	339,010	8,208	1,585
5 min	0.9	732,546	627,090	16,485	2,104
10 min	4.2	3,465,029	3,071,143	62,802	3,719
20 min	4.3	3,935,584	3,274,357	84,407	4,319

RNase A

cDNA Library	Amount** (nM cDNA)	# of Reads (Total)	HCV Specific Reads	UMI-Corrected HCV Reads	Cleavage Sites in HCV RNA
No RNase A	0.5	328,104	301,356	17,722	n/a
0 min	1.4	988,248	932,294	28,220	2,589
2.5 min	2.5	2,349,609	2,235,215	229,362	5,711
5 min	1.3	1,564,070	1,489,561	84,185	2,822
10 min	2.0	2,044,952	1,902,107	196,514	5,166
20 min	2.5	2,597,853	2,331,090	283,611	6,228

• RNA samples shown in Figure 1 used to make cDNA libraries.

**10 nM of cDNA, from multiplexed libraries, was used for each sequencing reaction.

Table S2. 2’, 3’-Cyclic Phosphate cDNA Synthesis and Illumina Sequencing: PV RNA* Cleaved by RNase L and RNase A.

RNase L					
cDNA Library	Amount** (nM cDNA)	# of Reads (Total)	PV Specific Reads	UMI-Corrected PV Reads	Cleavage Sites in PV RNA
0 min	2.0	5,085,075	4,713,577	22,918	2,432
2.5 min	2.0	5,689,034	5,357,623	39,148	2,920
5 min	2.0	8,016,229	7,574,667	93,092	4,112
10 min	2.0	7,396,964	6,969,182	102,237	4,222
20 min	2.0	7,532,038	7,193,692	161,147	4,639

RNase A					
cDNA Library	Amount** (nM cDNA)	# of Reads (Total)	PV Specific Reads	UMI-Corrected PV Reads	Cleavage Sites in PV RNA
No RNase A	0.03	4,624	2,904	122	n/a
0 min	0.07	10,086	9,304	624	343
2.5 min	1.4	846,279	817,960	27,274	1,786
5 min	3.7	2,048,956	1,944,899	68,006	3,296
10 min	2.2	1,168,582	1,086,158	62,903	3,120
20 min	1.8	982,942	889,833	43,317	2,735

•RNA samples shown in Figure 1 used to make cDNA libraries.

**10 nM of cDNA, from multiplexed libraries, was used for each sequencing reaction.

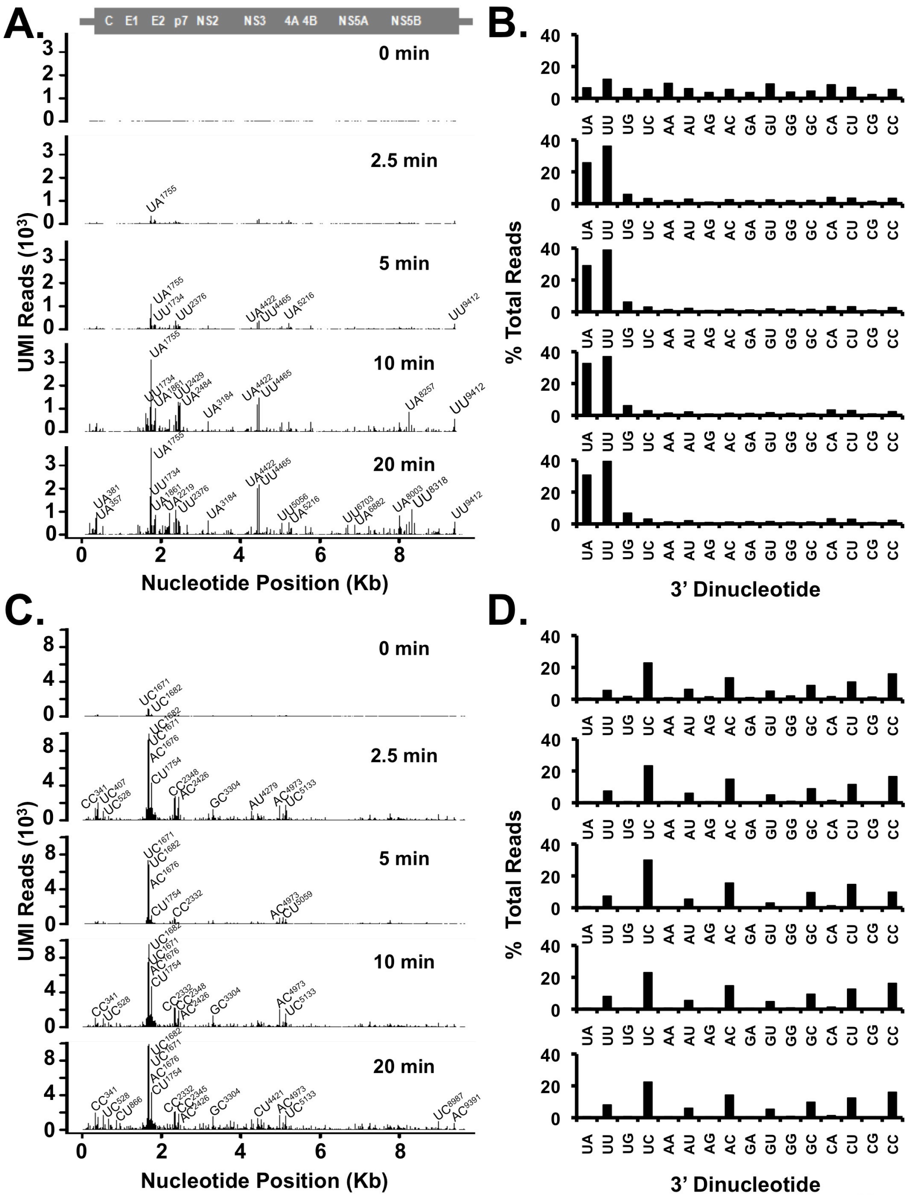

Figure S2. Endoribonuclease cleavage sites in HCV RNA detected using 2', 3'-cyclic phosphate cDNA synthesis and Illumina sequencing methods. The HCV RNAs in Figure 1 of the manuscript were used for 2', 3'-cyclic phosphate cDNA synthesis and Illumina sequencing. The amounts of cDNA sequenced for each sample are shown in Table S1.

A. Location and frequency of RNase L cleavage sites in HCV RNA. X-axis: Nucleotide position in HCV RNA. Y-axis: Number of distinct UMI linkers detected at each cleavage site. The 8 base UMI sequence in RNA linkers corresponds to 65,536 distinct linker sequences.

B. Dinucleotide specificity of RNase L cleavage sites in HCV RNA. X-axis: Dinucleotide at the 3' end of HCV RNA fragments (adjacent to 8 base UMI sequence in RNA linkers as illustrated in Figure S1). Y-axis: Percent of total cDNA reads.

C. Location and frequency of RNase A cleavage sites in HCV RNA. X-axis: Nucleotide position in HCV RNA. Y-axis: Number of distinct UMI linkers detected at each cleavage site.

D. Dinucleotide specificity of RNase A cleavage sites in HCV RNA. X-axis: Dinucleotide at the 3' end of HCV RNA fragments (adjacent to 8 base UMI sequence in RNA linkers as illustrated in Figure S1). Y-axis: Percent of total cDNA reads.

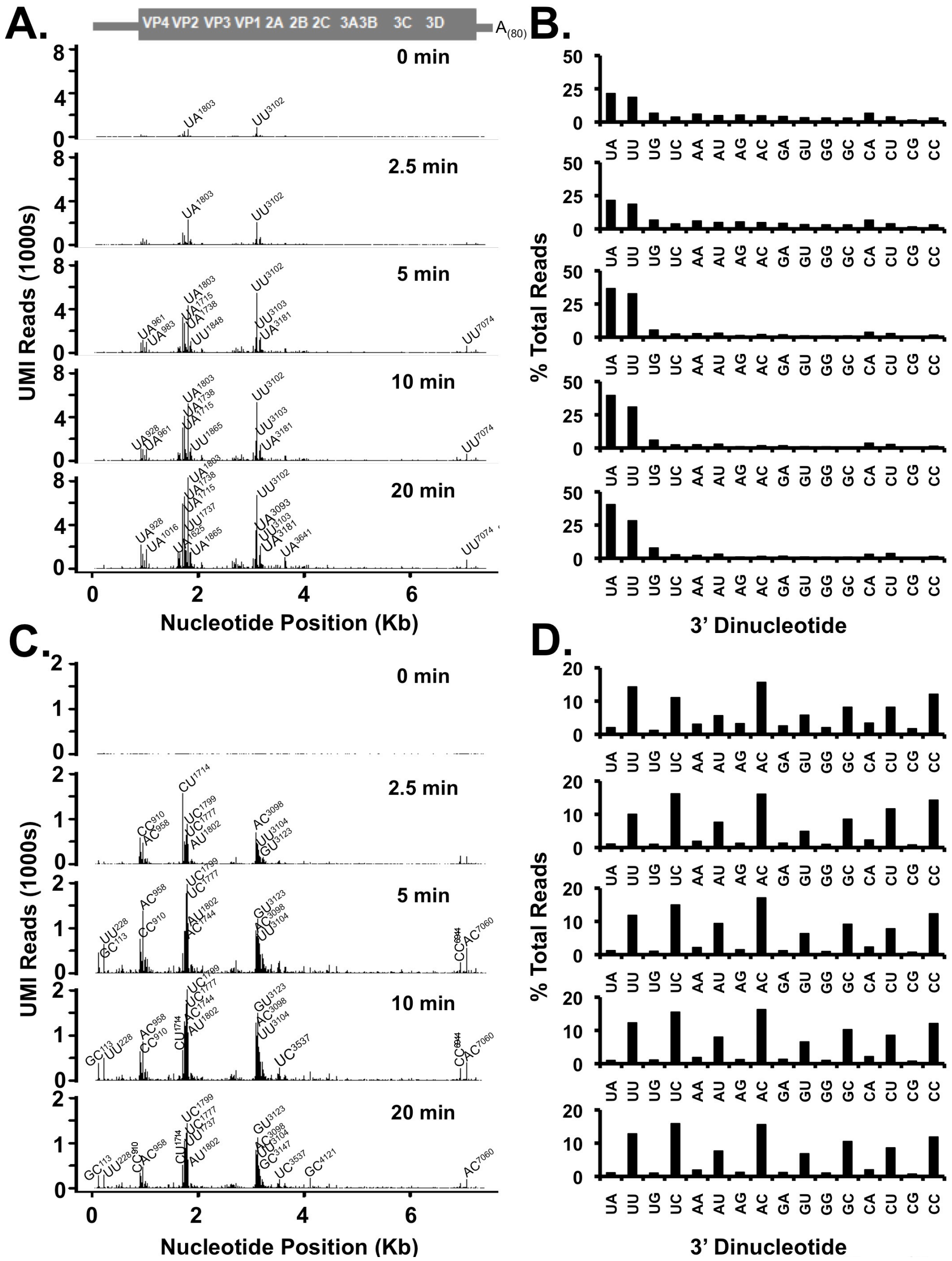

Figure S3. Endoribonuclease cleavage sites in PV RNA detected using 2', 3'-cyclic phosphate cDNA synthesis and Illumina sequencing methods. The PV RNAs shown in Figure 1 were used for 2', 3'-cyclic phosphate cDNA synthesis and Illumina sequencing. The amounts of cDNA sequenced for each sample are shown in Table S2.

A. **Location and frequency of RNase L cleavage sites in PV RNA.** X-axis: Nucleotide position in PV RNA. Y-axis: Number of distinct UMI linkers detected at each cleavage site.

B. **Dinucleotide specificity of RNase L cleavage sites in PV RNA.** X-axis: Dinucleotide at the 3' end of PV RNA fragments (adjacent to 8 base UMI sequence in RNA linkers as illustrated in Figure S1). Y-axis: Percent of total cDNA reads.

C. **Location and frequency of RNase A cleavage sites in PV RNA.** X-axis: Nucleotide position in PV RNA. Y-axis: Number of distinct UMI linkers detected at each cleavage site.

D. **Dinucleotide specificity of RNase A cleavage sites in PV RNA.** X-axis: Dinucleotide at the 3' end of PV RNA fragments (adjacent to 8 base UMI sequence in RNA linkers as illustrated in Figure S1). Y-axis: Percent of total cDNA reads.

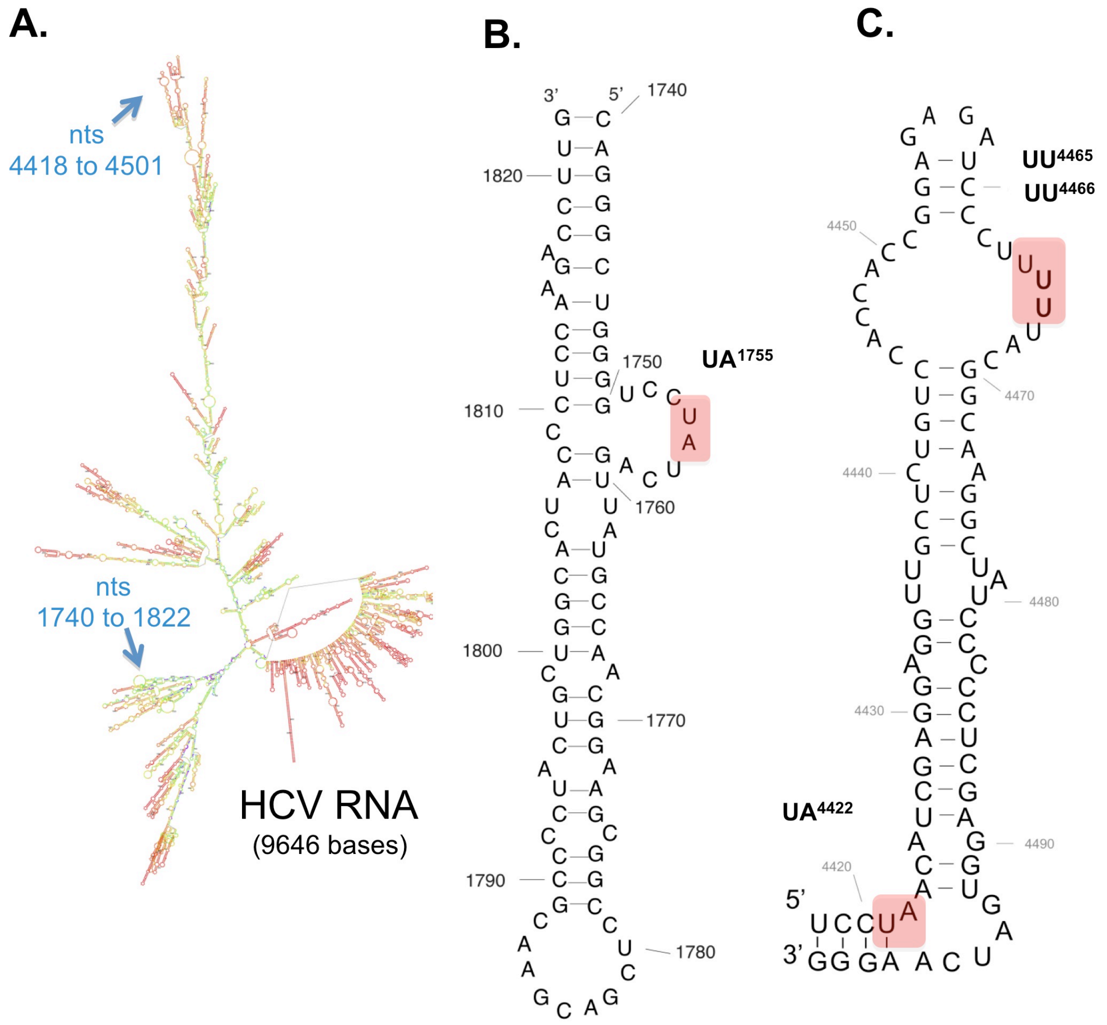

Figure S4. HCV RNA Secondary Structures Associated with RNase L Cleavage Sites.

A. HCV genotype 1a RNA secondary structure (NC_004102.1) determined by M-fold (3). The MinE structure of HCV is represented. The colors are bases with low (red), medium yellow) and high (purple) P-num values, as described previously (4). Image provided by A.C. Palmenberg.

B. HCV RNA nucleotides 1740 to 1822.

C. HCV RNA nucleotides 4418 to 4477.

D. Number of UMI-corrected cDNA reads at specific positions in HCV RNA.

D.

	cDNA Reads (UMI-Corrected)			
**Min**	**UA1755**	**UA4422**	**UU4465**	**UU4466**
**0**	0	0	0	0
**2.5**	284	130	180	82
**5**	986	256	332	148
**10**	2961	1070	1407	515
**20**	3579	1916	2113	896

A.

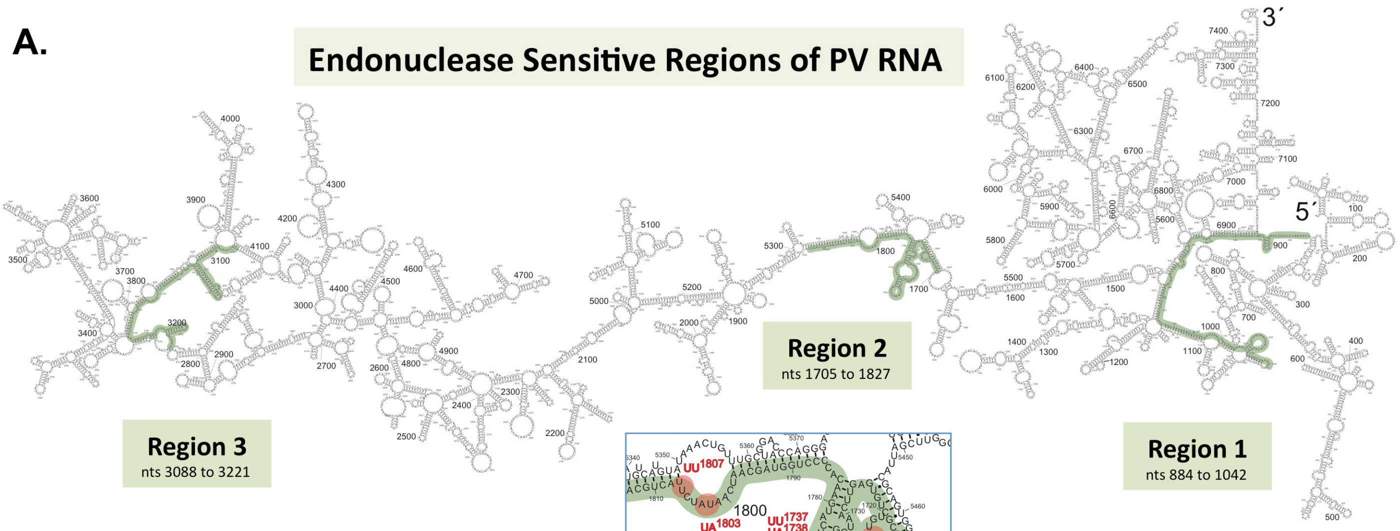

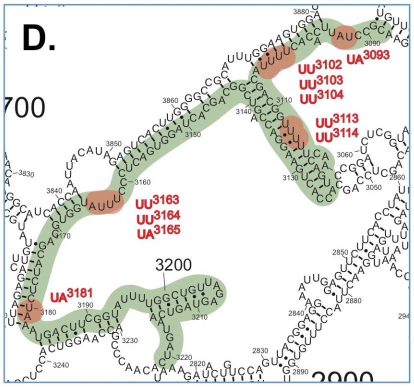

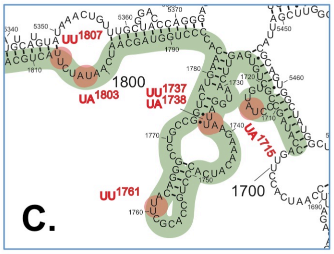

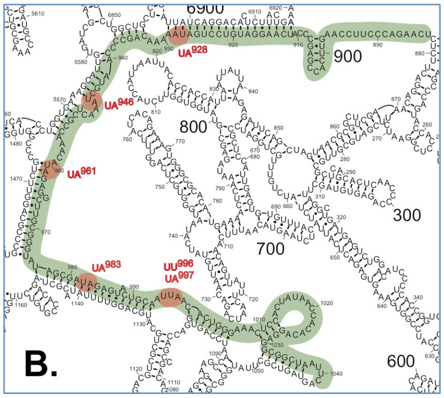

E. RNase L (5 min)

Base (nt)	UMI Reads
UA^{3093}	1367
UU^{3102}	5347
UU^{3103}	2517
UU^{3104}	941
UU^{3113}	559
UU^{3114}	433
UU^{3163}	1149
UU^{3164}	500
UA^{3165}	176
UA^{3181}	1391

Base (nt)	UMI Reads	Base (nt)	UMI Reads
UA^{1715}	3200	UA^{928}	880
UU^{1737}	1787	UA^{946}	161
UA^{1738}	2608	UA^{961}	1108
UU^{1761}	812	UA^{983}	523
UA^{1803}	4223	UU^{996}	523
UU^{1807}	1373	UA^{997}	460

Figure S5. Endoribonuclease susceptible regions of PV RNA.

A. Secondary structure of PV RNA. Endonuclease susceptible regions 1, 2, and 3 (green). M-fold data provided by A.C. Palmenberg and imaged using R2R (5).

B. Region 1 (RNase L cleavage sites highlighted).

C. Region 2 (RNase L cleavage sites highlighted).

D. Region 3 (RNase L cleavage sites highlighted).

E. Number of UMI-corrected cDNA reads at specific positions in PV RNA after 5 minutes of incubation with RNase L.

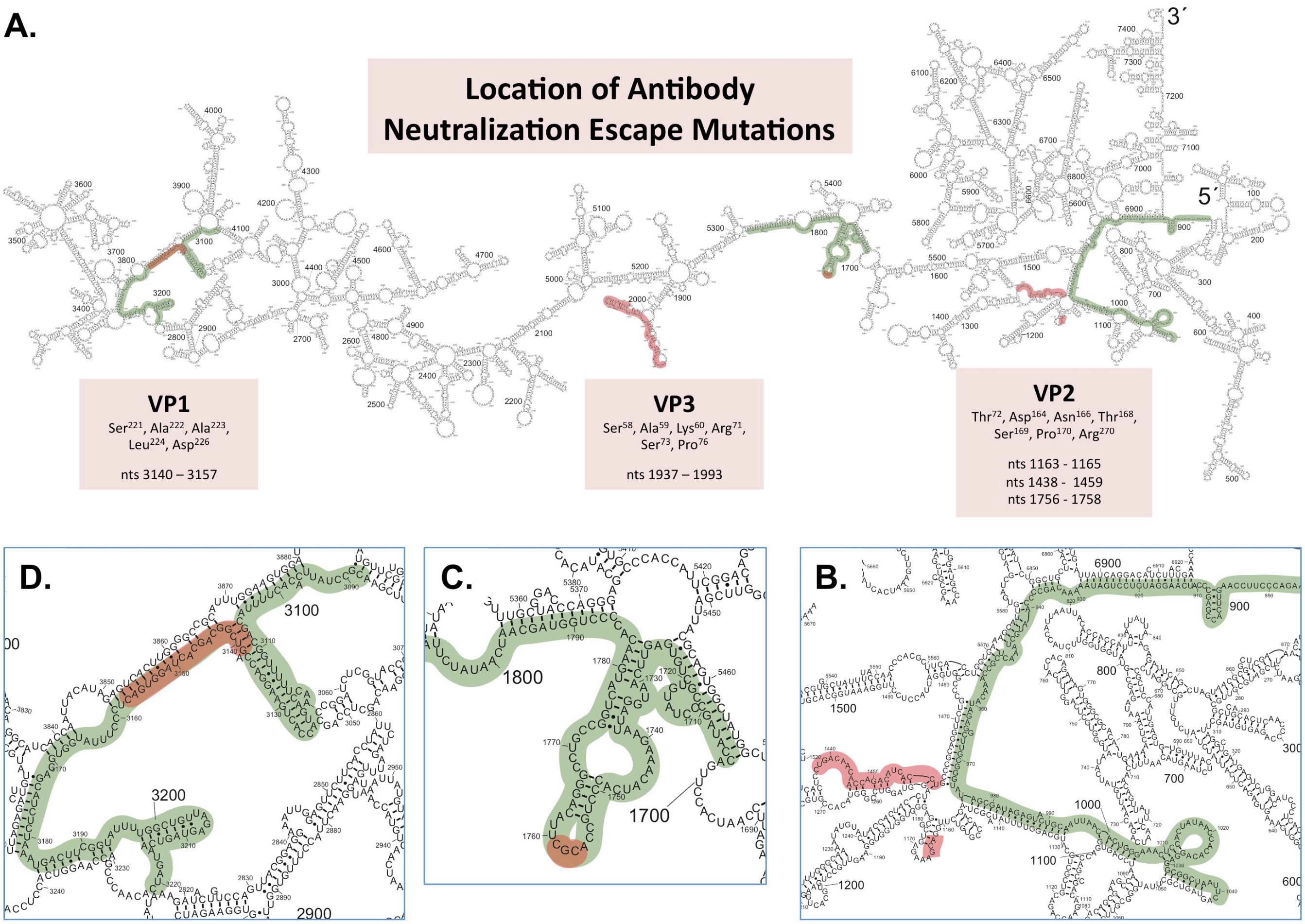

Figure S6. Antibody neutralization escape mutations within and near endonuclease susceptible regions of PV RNA.

A. Locations of antibody neutralization escape mutations in poliovirus RNA (pink). Capsid protein epitopes and corresponding neutralization escape mutations reported by Page et al., (6).

B. Region 1 (epitopes in VP2 are adjacent to endonuclease susceptible region 1 of PV RNA).

C. Region 2 [an epitope in VP2 (Arg^{270} / nts 1756 – 1758) maps within endonuclease susceptible region 2 of PV RNA].

D. Region 3 (neutralization epitopes in VP1 map within endonuclease susceptible region 3 of PV RNA).

Table S3. Host and Viral RNAs* with 2', 3'-Cyclic Phosphates

W12 HeLa Cells (wt RNase L)

	Mock 0 hpa	Mock 8 hpa	PV 0 hpa	PV 2 hpa	PV 4 hpa	PV 6 hpa	PV 8 hpa
**cDNA Reads**	1,114,940	1,079,935	1,773,129	2,220,324	928,770	801,016	1,026,829
**Polio (%)**	0.0	0.0	0.2	0.7	1.3	5.0	3.3
**Human (%)**	78.6	70.0	61.0	54.1	89.0	86.6	88.6
**Unaligned (%)**	21.4	30.0	38.8	45.2	9.7	8.4	8.1
**Total (%)**	100.0	10.00	100.0	100.0	100.0	10.00	100.0
**Most Frequent Human cDNA Reads (% Total Reads)**							
**28S rRNA**	15.3	11.1	18.2	14.7	22.3	18.8	26.6
**18S rRNA**	14.9	12.9	28.8	29.9	38.1	30.2	32.1
**5.8S rRNA**	1.9	1.1	0.3	0.2	0.5	0.5	0.1
**5S rRNA**	24.8	13.8	8.3	3.9	17.2	29.9	25.4
**U6 snRNA**	10.9	14.8	2.4	2.8	6.0	3.2	1.2
**mRNA & other**	10.8	16.3	3.0	2.6	4.9	4.0	3.2

M25 HeLa Cells (DN RNase L)

	Mock 0 hpa	Mock 8 hpa	PV 0 hpa	PV 2 hpa	PV 4 hpa	PV 6 hpa	PV 8 hpa
**cDNA Reads**	1,209,291	1,212,835	1,321,278	1,478,313	1,612,231	1,200,655	1,718,384
**Polio (%)**	0.0	0.0	0.0	0.0	9.4	26.1	2.6
**Human (%)**	74.8	64.3	79.0	69.1	75.0	60.8	57.9
**Unaligned (%)**	25.2	35.7	21.0	30.9	15.6	13.1	39.5
**Total (%)**	100.0	10.00	100.0	100.0	100.0	10.00	100.0
**Most Frequent Human cDNA Reads (% Total Reads)**							
**28S rRNA**	15.4	12.3	23.9	15.7	21.3	17.3	8.1
**18S rRNA**	13.9	13.4	20.2	14.7	16.8	17.5	8.7
**5.8S rRNA**	1.8	0.9	1.8	1.5	1.3	0.8	1.9
**5S rRNA**	24.9	13.1	16.4	22.9	12.1	9.3	27.3
**U6 snRNA**	8.5	9.6	10.6	10.0	12.4	5.8	8.4
**mRNA & other**	10.3	15.0	6.1	4.3	11.1	10.1	3.5

* RNA samples shown in Figure 3B used to make cDNA libraries.

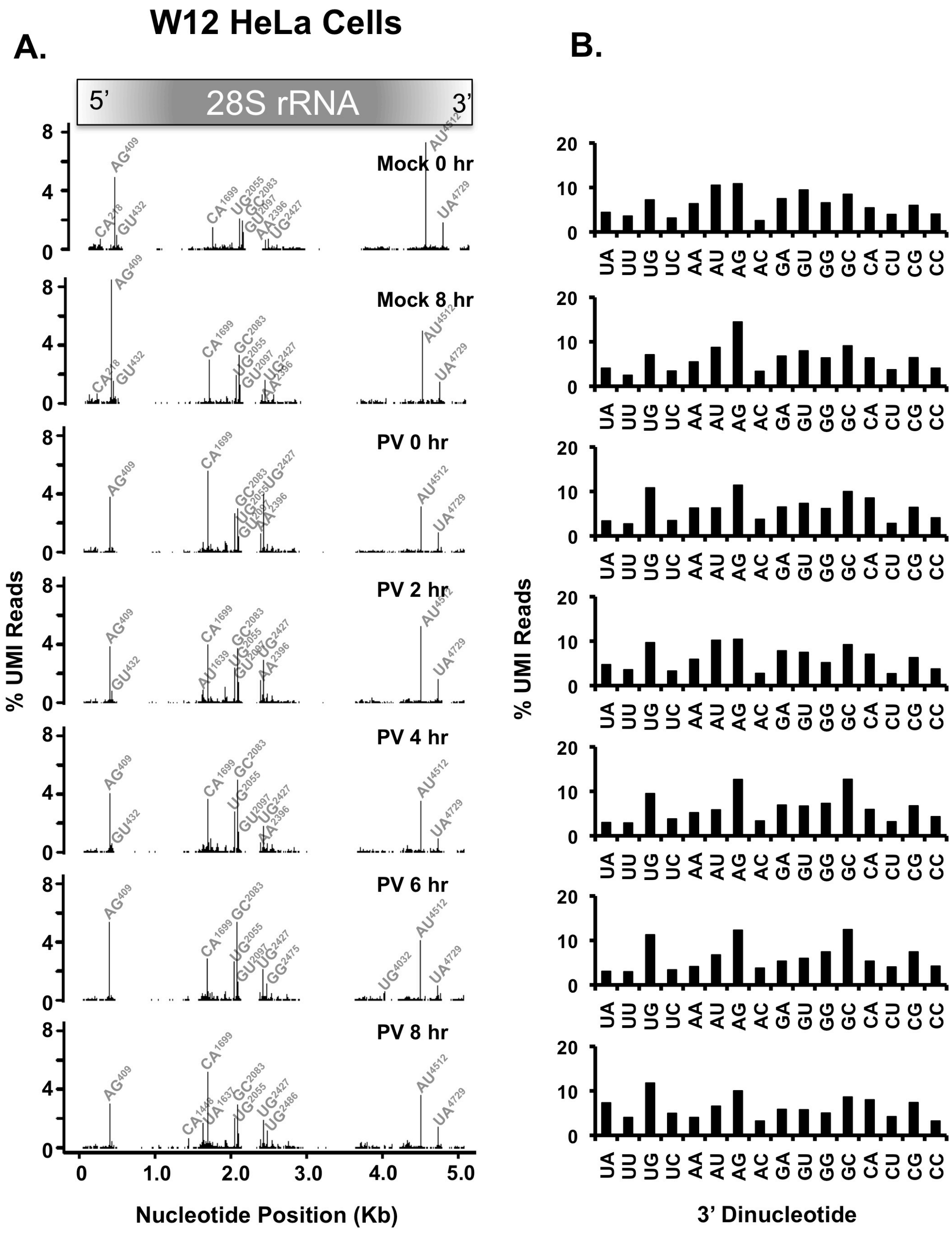

Figure S7. Frequency, location and dinucleotide specificity of endoribonuclease cleavage sites in 28S rRNA from W12 HeLa cells. RNAs from mock-infected and PV-infected W12 HeLa cells were used for 2', 3'-cyclic phosphate cDNA synthesis and Illumina sequencing.

A. The location and frequency of cleavage sites in 28S rRNA. X-axis: Nucleotide position of 28S rRNA. Y-axis: % of total UMIs in 28S rRNA. Dinucleotides at the 3' end of abundant RNA fragments are annotated at the corresponding positions in the graphs.

B. Dinucleotides at the 3' end of 28S rRNA fragments. X-axis: Dinucleotide at the 3' end of 28S rRNA fragments. Y-axis: Percent of total cDNA reads in 28S rRNA.

Figure S7
Cooper et al.

M25 HeLa Cells

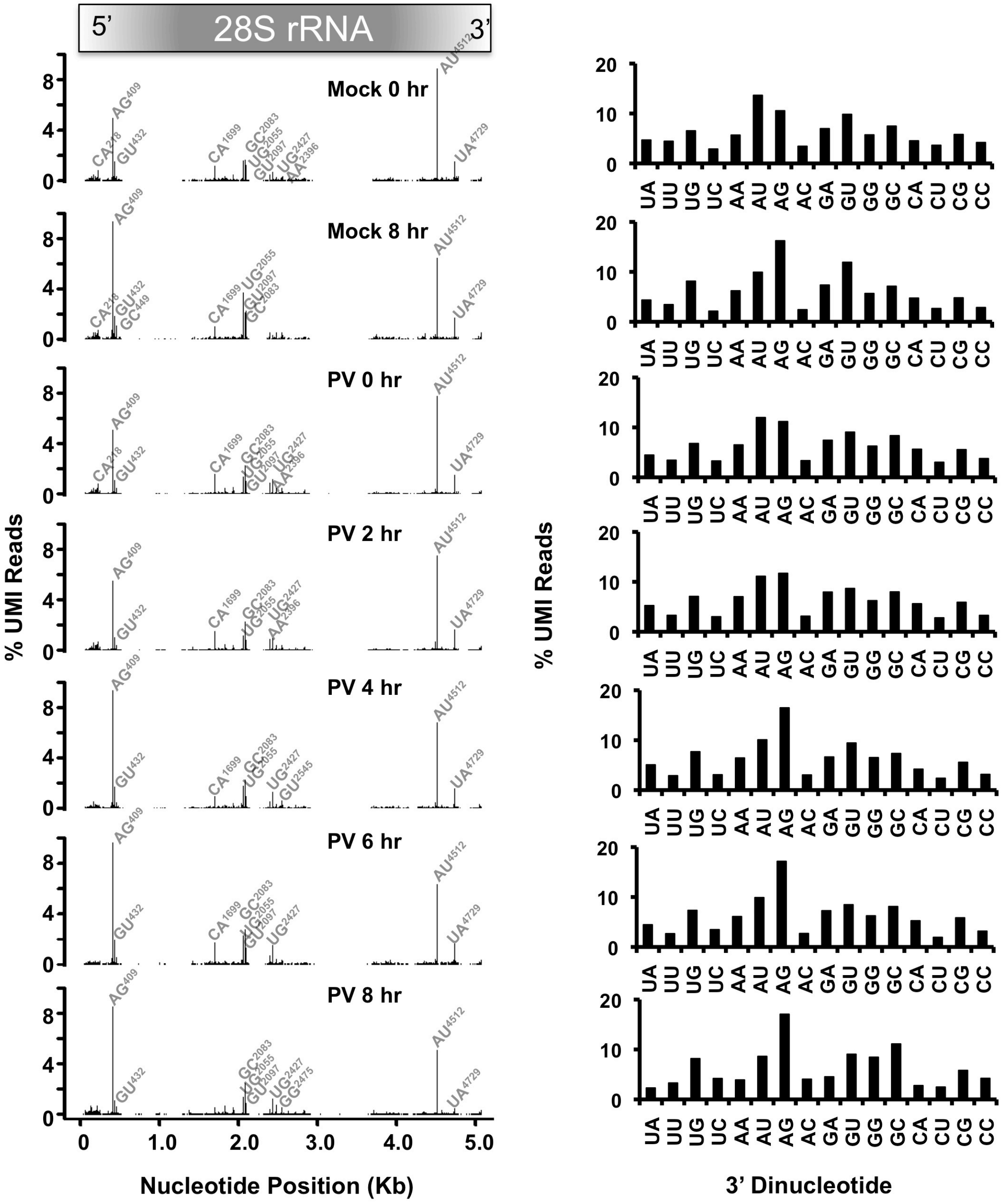

Figure S8. Frequency, location and dinucleotide specificity of endoribonuclease cleavage sites in 28S rRNA from M25 HeLa cells. RNAs from mock-infected and PV-infected M25 HeLa cells were used for 2', 3'-cyclic phosphate cDNA synthesis and Illumina sequencing.

A. The location and frequency of cleavage sites in 28S rRNA. X-axis: Nucleotide position of 28S rRNA. Y-axis: % of total UMIs in 28S rRNA. Dinucleotides at the 3' end of abundant RNA fragments are annotated at the corresponding positions in the graphs.

B. Dinucleotides at the 3' end of 28S rRNA fragments. X-axis: Dinucleotide at the 3' end of 28S rRNA fragments. Y-axis: Percent of total cDNA reads in 28S rRNA.

Figure S8
Cooper et al.

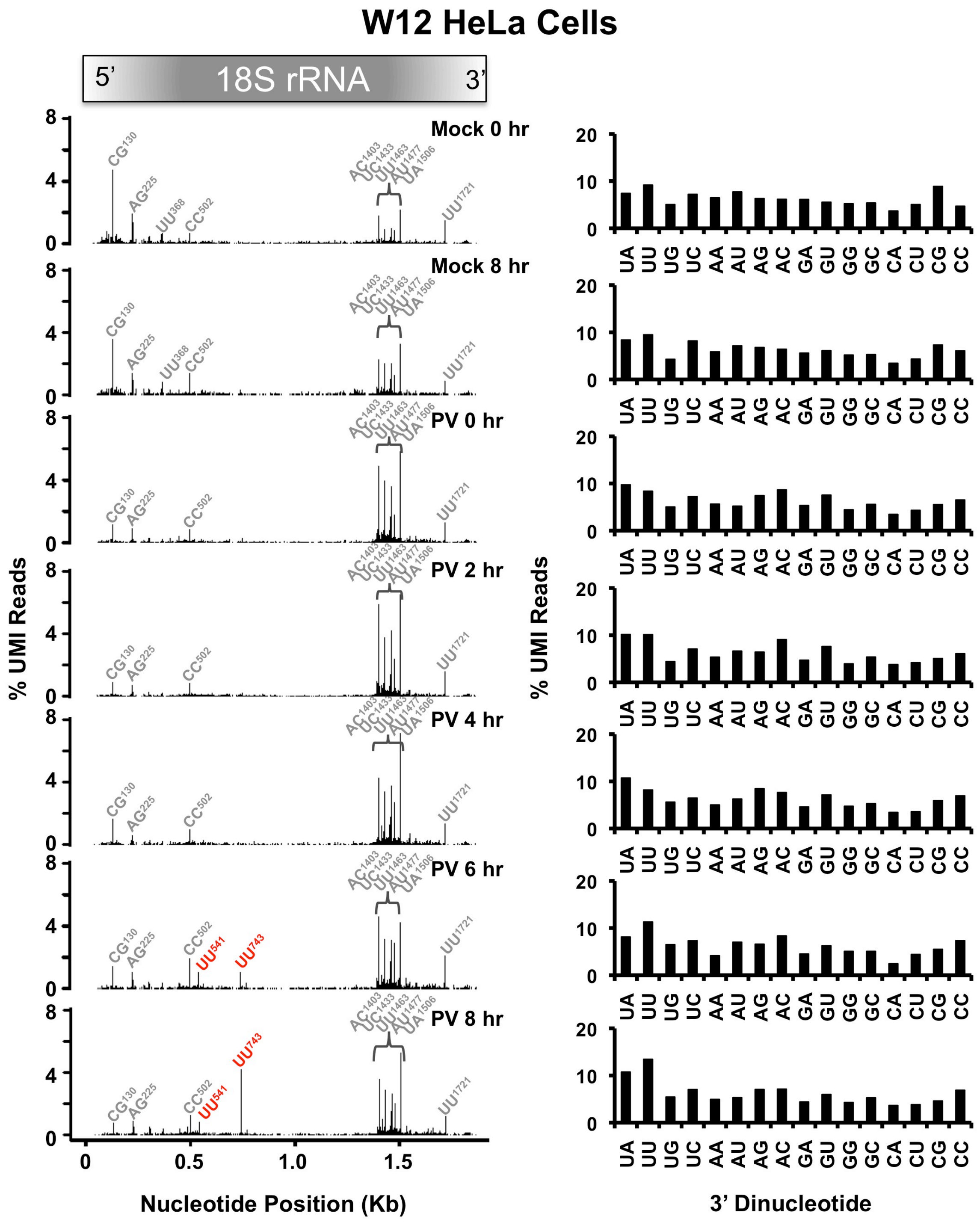

Figure S9. Frequency, location and dinucleotide specificity of endoribonuclease cleavage sites in 18S rRNA from W12 HeLa cells. RNAs from mock-infected and PV-infected W12 HeLa cells were used for 2', 3'-cyclic phosphate cDNA synthesis and Illumina sequencing.

A. The location and frequency of cleavage sites in 18S rRNA. Dinucleotides at the 3' end of abundant RNA fragments are annotated at the corresponding positions in the graphs. RNase L specific cleavage sites are highlighted in red.

B. Dinucleotides at the 3' end of 18S rRNA fragments.

M25 HeLa Cells

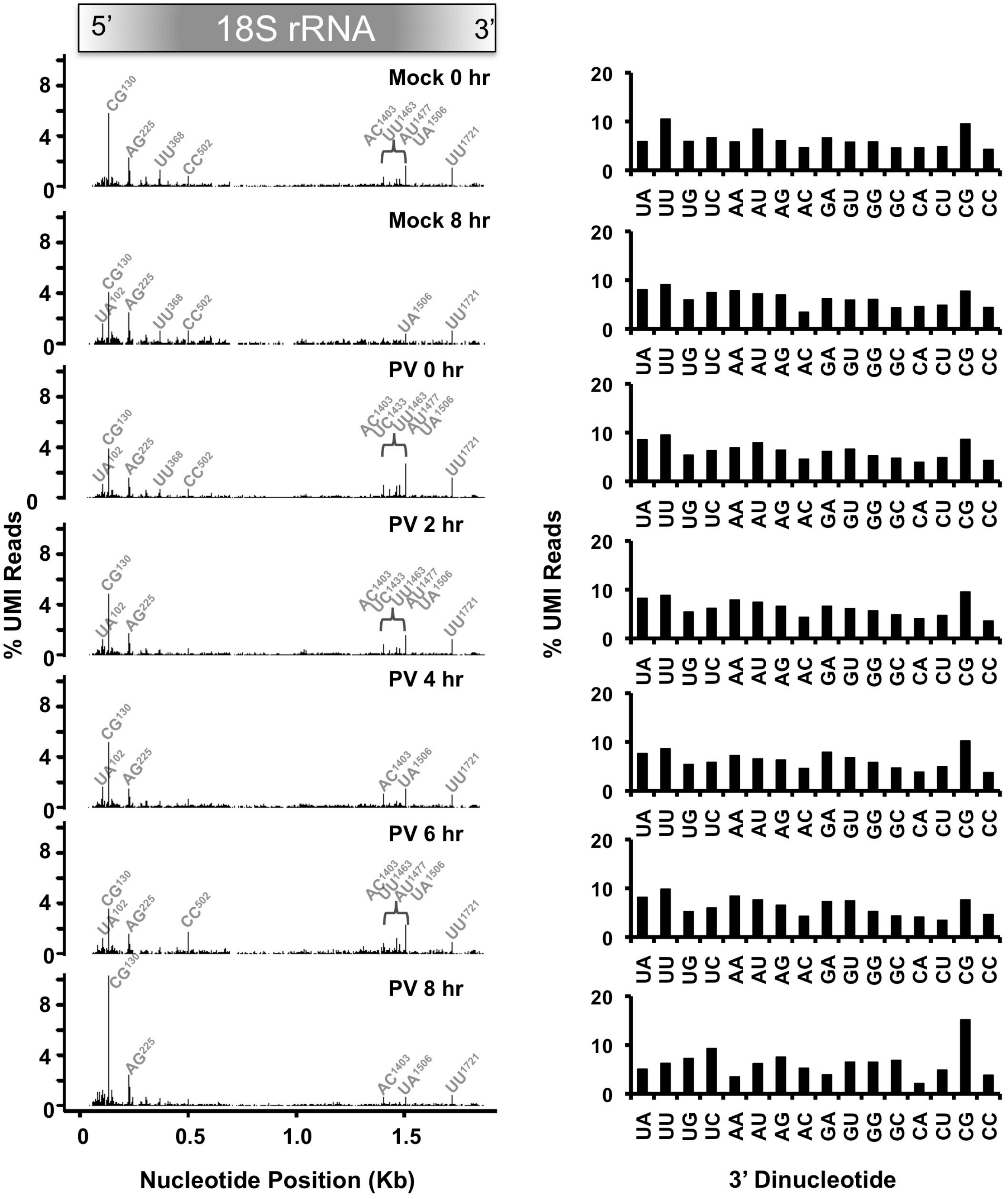

Figure S10. Frequency, location and dinucleotide specificity of endoribonuclease cleavage sites in 18S rRNA from M25 HeLa cells. RNAs from mock-infected and PV-infected M25 HeLa cells were used for 2', 3'-cyclic phosphate cDNA synthesis and Illumina sequencing.

A. Location and frequency of cleavage sites in 18S rRNA. Dinucleotides at the 3' end of abundant RNA fragments are annotated at the corresponding positions in the graphs.

B. Dinucleotides at the 3' end of 18S rRNA fragments.

Figure S10
Cooper et al.

5.8S rRNA

A.

```
5′ cgacucuuag cgguggauca cucggcucgu gcgucgauga agaacgcagc uagcugcgag60
   aauuaaugug aauugcagga cacauugauc aucgacacuu cgaacgcacu ugcggccccg120
   gguuccuccc ggggcuacgc cugucugagc gucgcu156u  3′
```

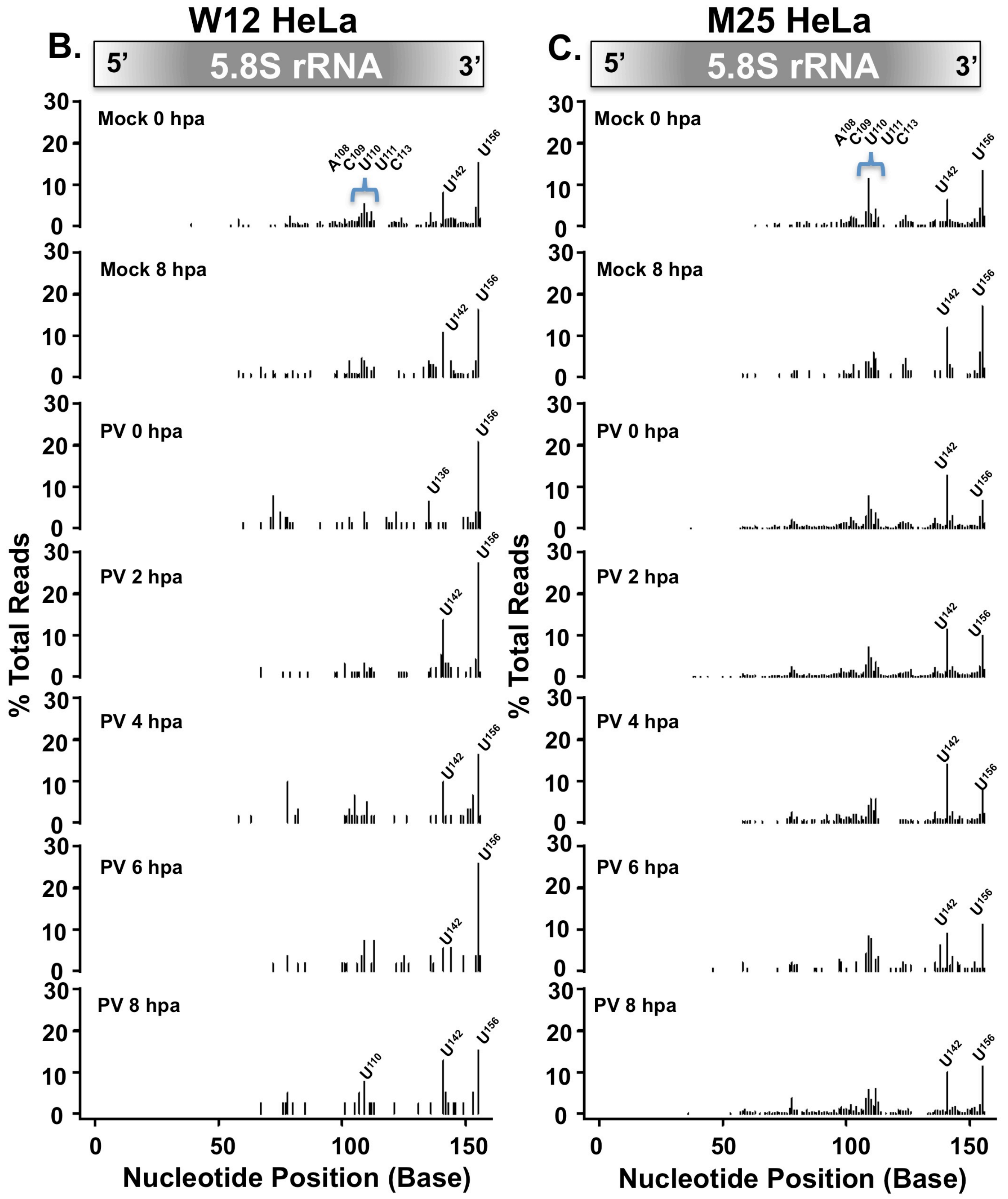

Figure S11. Frequency and location of 2’, 3’-cyclic phosphates in 5.8S rRNA. A. 5.8S rRNA (NCBI # NR_003285.2). 5’-terminal C residue added to match sequence from Anger et al., 2013 (7). Sites with 2’, 3’-cyclic phosphates highlighted in red. 5.8S rRNA from W12 (B) and M25 HeLa cells (C).

5S rRNA

A. 5′ gucuacggcc[10] auaccacccu[20] gaacgcgccc[30] gaucucgucu[40] gaucucggaa[50]
gcuaagcagg[60] gucgggccug[70] guuaguacuu[80] ggacgggaga[90] ccgccuggga[100]
auaccgggug[110] cuguaggcuu[120] u 3′

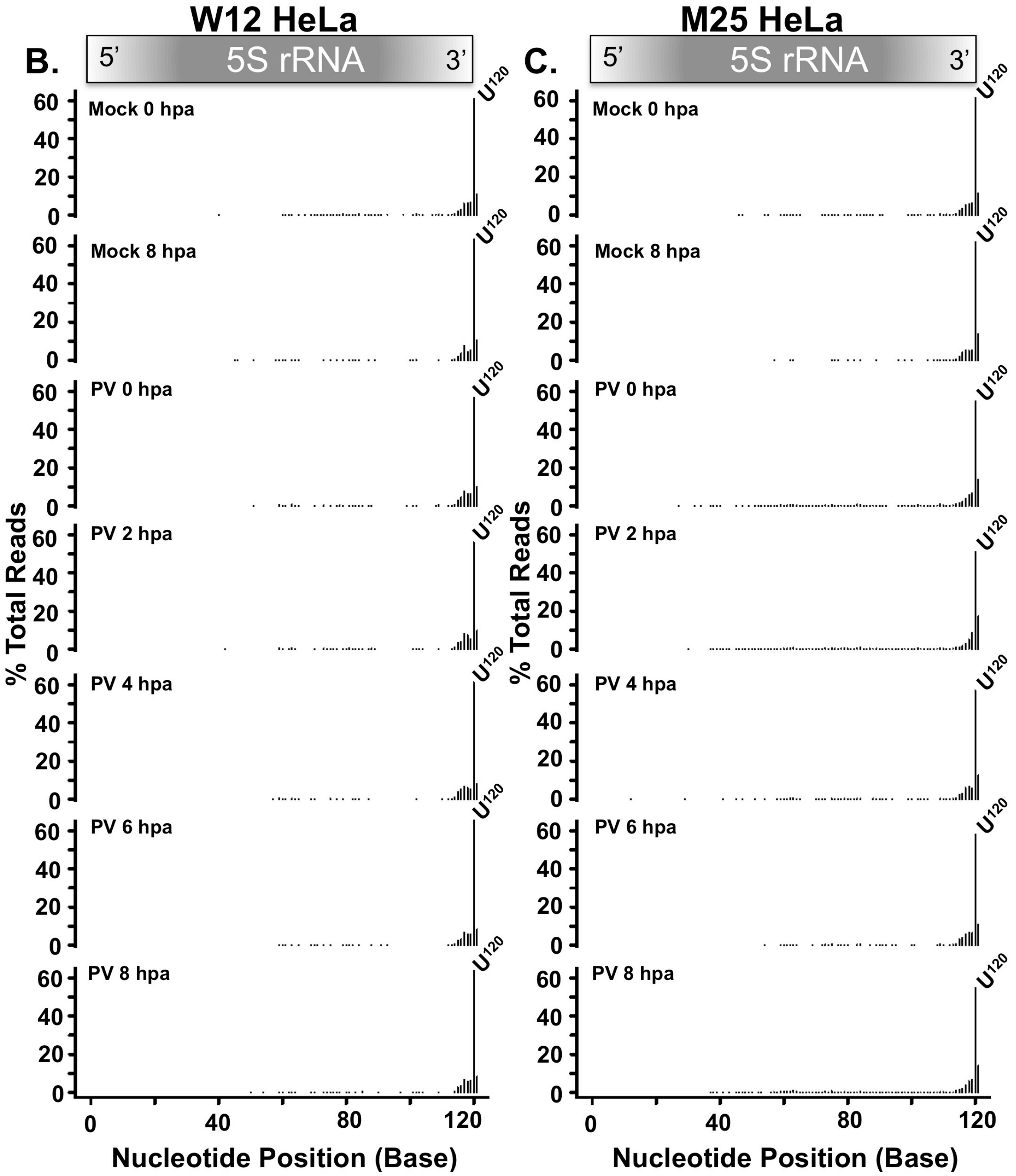

Figure S12. Frequency and location of 2’, 3’-cyclic phosphates in 5S rRNA.
A. 5S rRNA (NCBI # NR_023371.1). Sites with 2’, 3’-cyclic phosphates highlighted in red.
B. 5S rRNA from W12 HeLa cells.
C. 5S rRNA from M25 HeLa cells.

Figure S12
Cooper et al.

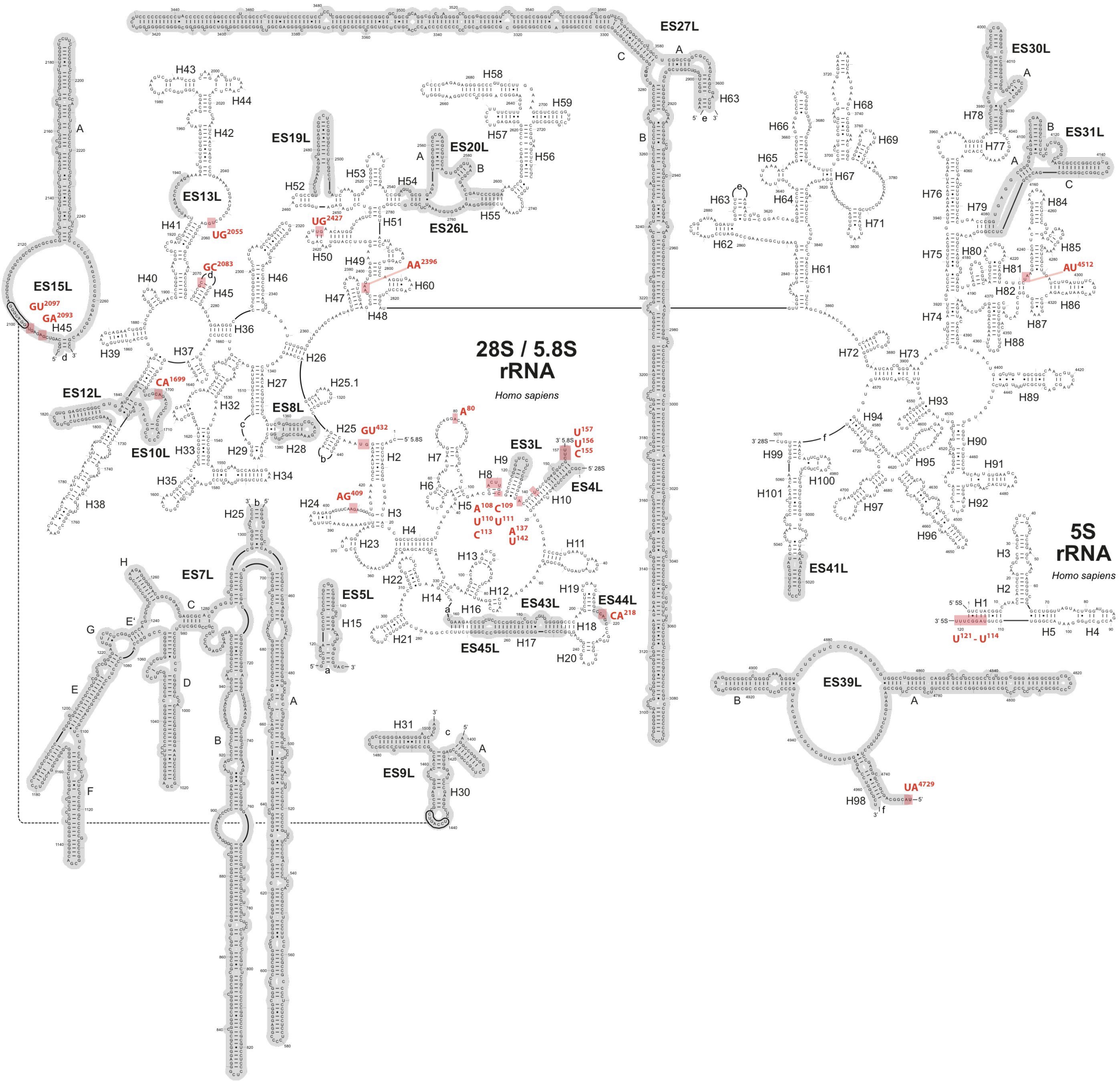

Figure S13. Location of common cleavage sites in the RNA secondary structures of 60S subunit rRNAs (28S / 5.8S / 5S). rRNA secondary structures from Anger et al., 2013 (7) modified to show locations of endonuclease cleavage.

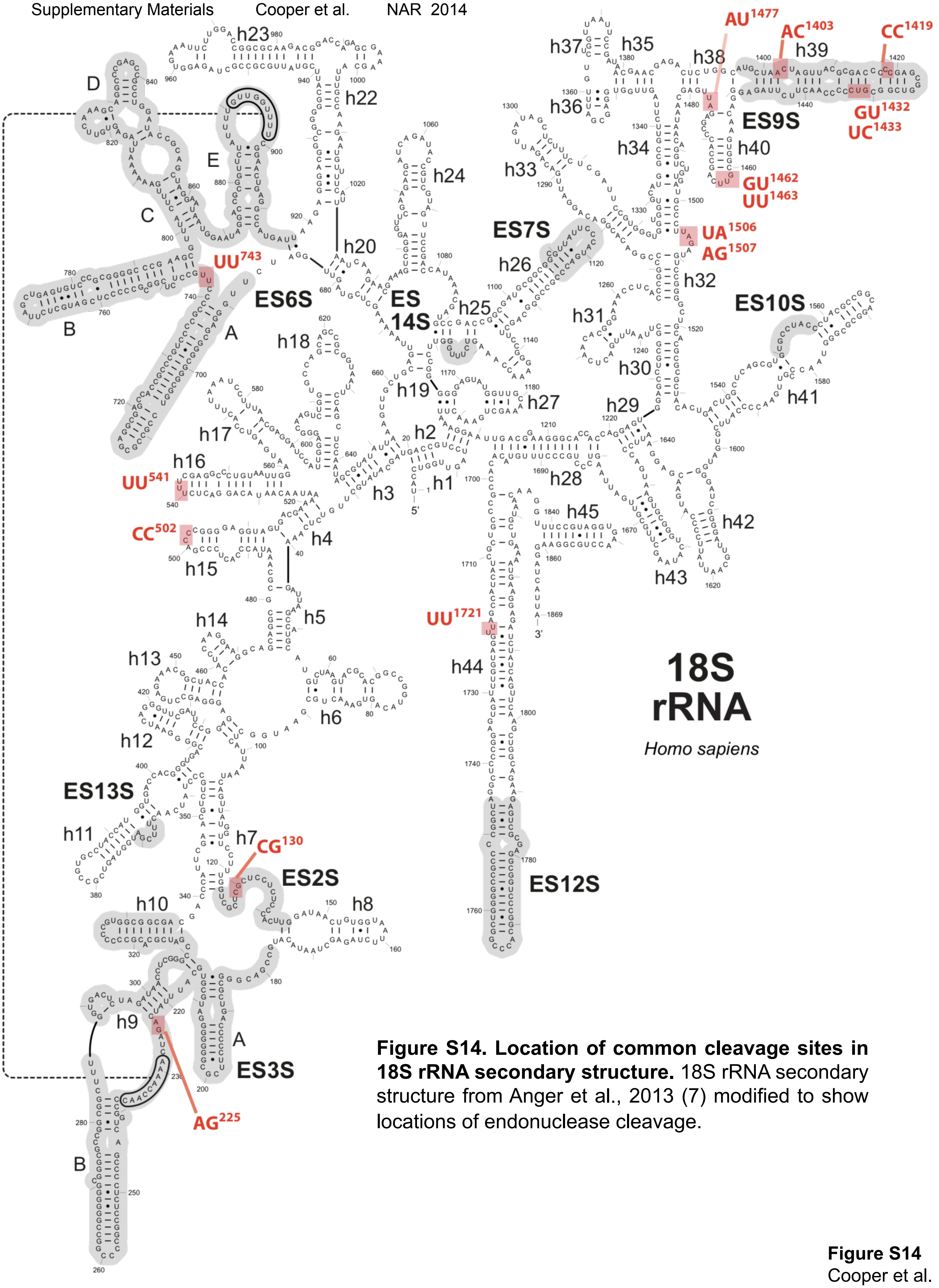

Figure S14. Location of common cleavage sites in 18S rRNA secondary structure. 18S rRNA secondary structure from Anger et al., 2013 (7) modified to show locations of endonuclease cleavage.

A.

U6 snRNA

```
5′ gugcucgcuu10 cagcagcaca20 uauacuaaaa30 uacgaacgau40
   acagagaaga50 uuagcauggc60 cccugcgcaa70 ggaugacacg80
   caaauucgug90 aagcguucca100 uauuuuuu 3′
```

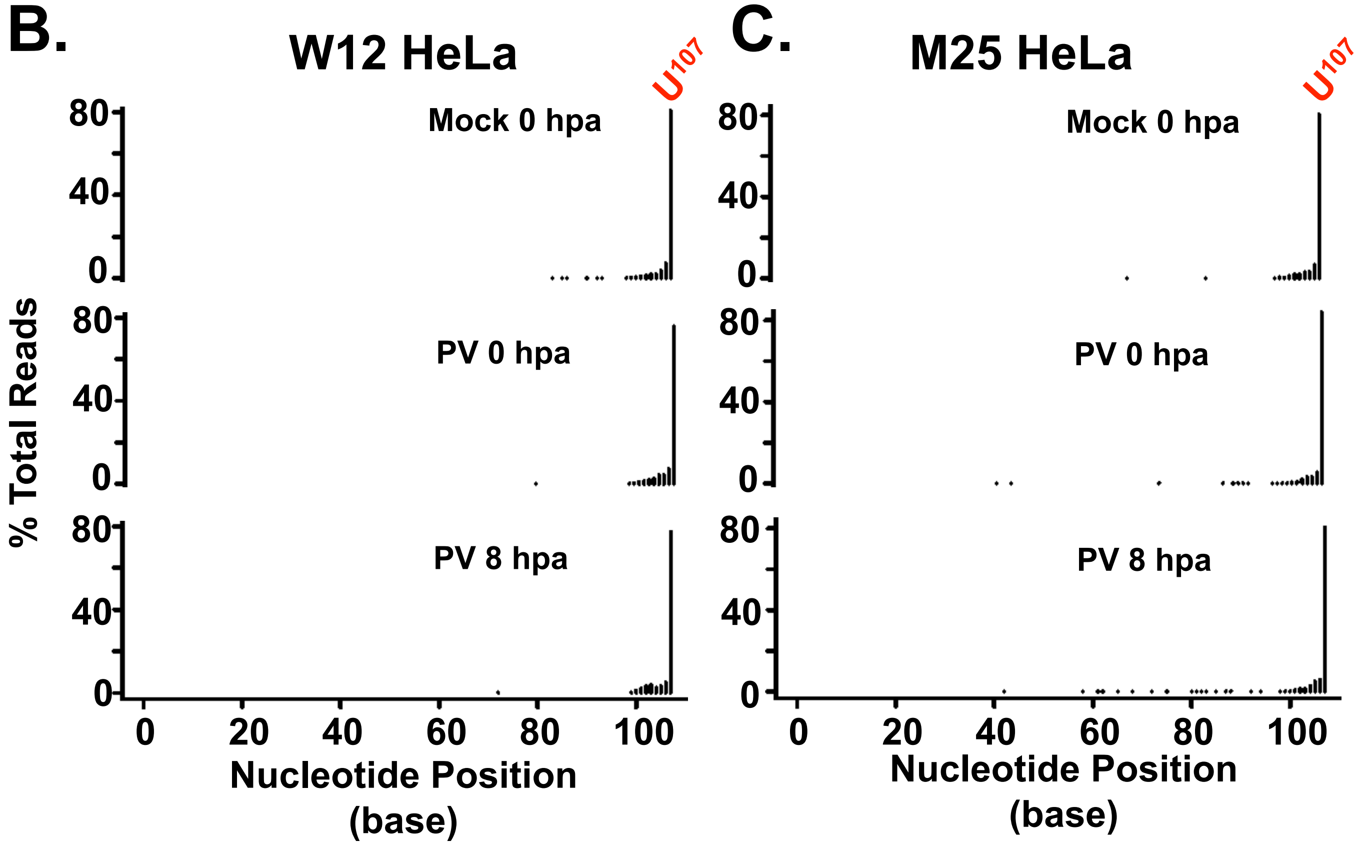

Figure S15. U6 snRNA. Sequence of U6 snRNA **(A)**. Location and frequency of cleavage sites in U6 snRNA from W12 HeLa cells **(B)** and M25 HeLa cells **(C)**. The 2’, 3’-cyclic phosphates at the end of U6 snRNA are consistent with the enzymatic activity of C16orf57 (8,9).

Table S4. Common cleavage sites in 28S rRNA

W12 HeLa Cells / Cleavage Location & Frequency in 28S rRNA (% of 28S UMI Reads)							
Location in 28S rRNA	Mock 0 hr	Mock 8 hr	PV 0 hpa	PV 2 hpa	PV 4 hpa	PV 6 hpa	PV 8 hpa
CA^{218}	0.7	0.7	0.2	0.3	0.1	0.1	0.0
AG^{409}	4.9	8.3	3.7	3.8	4.0	5.4	3.1
GU^{432}	1.0	1.6	0.4	0.8	0.7	0.4	0.4
CA^{1699}	1.5	3.1	5.6	4.0	3.6	2.7	5.2
UG^{2055}	2.1	1.9	2.6	2.4	2.8	2.6	2.1
GC^{2083}	2.0	3.3	3.0	3.7	5.0	5.3	2.9
GA^{2093}	0.6	0.5	0.8	1.2	0.8	0.7	0.7
GU^{2097}	1.2	1.3	1.1	1.4	1.4	1.2	1.0
AA^{2396}	0.6	0.6	1.3	1.6	0.7	0.6	0.6
UG^{2427}	0.7	1.6	3.7	2.8	1.8	2.1	1.9
AU^{4512}	7.3	5.0	3.2	5.3	3.5	4.2	3.7
UA^{4729}	1.8	1.4	1.4	1.6	1.0	1.0	1.4

M25 HeLa Cells / Cleavage Location & Frequency in 28S rRNA (% of 28S UMI Reads)							
Location in 28S rRNA	Mock 0 hr	Mock 8 hr	PV 0 hpa	PV 2 hpa	PV 4 hpa	PV 6 hpa	PV 8 hpa
CA^{218}	0.8	0.7	0.8	0.6	0.2	0.2	0.1
AG^{409}	4.9	9.4	5.1	5.5	9.5	9.8	8.5
GU^{432}	1.5	1.8	1.1	1.0	1.7	1.9	1.1
CA^{1699}	1.2	1.0	1.5	1.5	1.0	1.7	0.5
UG^{2055}	1.6	3.6	1.3	1.1	1.8	2.2	1.4
GC^{2083}	1.7	2.2	2.3	2.3	2.2	2.7	2.5
GA^{2093}	0.2	0.4	0.7	0.7	0.7	0.6	0.3
GU^{2097}	1.2	2.3	1.0	0.8	0.9	1.3	0.9
AA^{2396}	0.5	0.5	0.8	0.8	0.5	0.7	0.4
UG^{2427}	0.7	0.3	1.1	0.9	1.3	1.6	1.3
AU^{4512}	8.9	6.5	7.8	7.5	6.8	6.3	5.1
UA^{4729}	1.5	1.7	1.5	1.6	1.6	1.7	0.4

Table S5. Common cleavage sites in 18S rRNA

W12 HeLa Cells / Cleavage Location & Frequency in 18S rRNA (% of 18S UMI Reads)

Location in 18S rRNA	Mock 0 hr	Mock 8 hr	PV 0 hpa	PV 2 hpa	PV 4 hpa	PV 6 hpa	PV 8 hpa
CG^{130}	4.7	3.6	1.1	0.9	1.7	1.4	0.8
AG^{225}	1.9	1.4	0.8	0.7	0.6	1.0	0.9
CC^{502}	0.7	1.4	0.8	0.8	1.0	2.0	1.3
UU^{541}	0.0	0.1	0.1	0.0	0.1	1.0	0.8
**UU^{743}**	**0.1**	**0.3**	**0.1**	**0.1**	**0.1**	**1.1**	**4.3**
AC^{1403}	1.8	2.3	5.0	5.8	4.3	4.7	3.6
CC^{1419}	0.3	0.5	0.6	0.7	1.2	0.9	1.0
GU^{1432}	0.4	0.8	1.9	1.7	1.6	1.0	1.4
UC^{1433}	0.9	2.0	3.8	3.7	3.5	3.2	2.9
GU^{1462}	0.4	1.0	1.7	1.4	1.7	1.7	1.5
UU^{1463}	1.0	2.0	3.7	4.2	3.7	3.1	2.7
AU^{1477}	0.8	1.2	1.8	2.4	2.7	2.9	2.0
UA^{1506}	2.2	3.3	6.0	6.6	7.1	4.2	5.2
AG^{1507}	0.4	1.0	1.6	1.1	2.3	1.1	1.6
UU^{1721}	1.5	0.9	1.3	1.6	1.3	2.0	1.2

M25 HeLa Cells / Cleavage Location & Frequency in 18S rRNA (% of 18S UMI Reads)

Location in 18S rRNA	Mock 0 hr	Mock 8 hr	PV 0 hpa	PV 2 hpa	PV 4 hpa	PV 6 hpa	PV 8 hpa
CG^{130}	5.7	4.0	3.9	4.8	5.0	3.5	10.1
AG^{225}	2.3	2.4	1.5	1.7	1.4	1.6	2.5
CC^{502}	0.8	1.0	0.7	0.5	0.6	1.7	0.5
UU^{541}	0.0	0.0	0.0	0.0	0.0	0.0	0.0
**UU^{743}**	**0.1**	**0.0**	**0.1**	**0.1**	**0.1**	**0.0**	**0.0**
AC^{1403}	0.7	0.4	1.0	0.8	1.1	0.9	0.6
CC^{1419}	0.0	0.1	0.1	0.0	0.2	0.3	0.1
GU^{1432}	0.1	0.1	0.2	0.1	0.1	0.1	0.1
UC^{1433}	0.3	0.0	0.7	0.4	0.3	0.4	0.2
GU^{1462}	0.2	0.2	0.4	0.2	0.3	0.3	0.2
UU^{1463}	0.5	0.3	0.9	0.6	0.5	1.2	0.1
AU^{1477}	0.6	0.1	1.0	0.5	0.3	0.8	0.3
UA^{1506}	1.5	0.8	2.7	1.5	1.5	2.3	0.7
AG^{1507}	0.1	0.1	0.4	0.3	0.1	0.3	0.5
UU^{1721}	1.5	1.0	1.5	1.2	1.0	0.9	0.8

Table S6. Common cleavage sites in 5.8S rRNA

W12 HeLa Cells / Cleavage Location & Frequency in 5.8S rRNA (UMI Reads)

Location in 5.8S rRNA	Mock 0 hr	Mock 8 hr	PV 0 hpa	PV 2 hpa	PV 4 hpa	PV 6 hpa	PV 8 hpa
A^{80}	9	0	1	1	0	0	0
A^{108}	8	1	0	1	0	0	2
C^{109}	11	6	0	0	1	2	0
U^{110}	20	5	3	3	1	4	3
U^{111}	12	3	1	1	3	0	0
C^{113}	13	2	0	2	1	1	1
A^{137}	12	4	1	2	1	2	1
U^{142}	30	14	1	13	6	3	5
C^{155}	17	5	3	4	0	2	0
U^{156}	57	21	16	26	10	14	6
U^{157}	7	0	3	1	1	2	0

M25 HeLa Cells / Cleavage Location & Frequency in 5.8S rRNA (UMI Reads)

Location in 5.8S rRNA	Mock 0 hr	Mock 8 hr	PV 0 hpa	PV 2 hpa	PV 4 hpa	PV 6 hpa	PV 8 hpa
A^{80}	0	2	19	37	2	2	9
A^{108}	1	0	11	26	2	0	8
C^{109}	12	5	37	74	4	6	37
U^{110}	39	5	97	181	13	12	60
U^{111}	10	3	57	116	18	11	35
C^{113}	14	6	47	91	18	4	62
A^{137}	5	2	30	55	8	3	8
U^{142}	22	16	161	294	44	13	103
C^{155}	15	8	35	64	6	1	23
U^{156}	46	23	85	255	26	16	117
U^{157}	8	3	15	45	7	1	4

References

1. Schutz, K., Hesselberth, J.R. and Fields, S. (2010) Capture and sequence analysis of RNAs with terminal 2',3'-cyclic phosphates. *RNA*, **16**, 621-631.

2. Kivioja, T., Vaharautio, A., Karlsson, K., Bonke, M., Enge, M., Linnarsson, S. and Taipale, J. (2012) Counting absolute numbers of molecules using unique molecular identifiers. *Nat Methods*, **9**, 72-74.

3. Zuker, M. (2003) Mfold web server for nucleic acid folding and hybridization prediction. *Nucleic Acids Res*, **31**, 3406-3415.

4. Palmenberg, A.C. and Sgro, J.Y. (1997) Topological organization of picornaviral genomes: Statistical prediction of RNA structural signals. *Semin Virol*, **8**, 231-241.

5. Weinberg, Z. and Breaker, R.R. (2011) R2R--software to speed the depiction of aesthetic consensus RNA secondary structures. *BMC Bioinformatics*, **12**, 3.

6. Page, G.S., Mosser, A.G., Hogle, J.M., Filman, D.J., Rueckert, R.R. and Chow, M. (1988) 3-Dimensional structure of poliovirus serotype-1 neutralizing determinants. *J. Virol.*, **62**, 1781-1794.

7. Anger, A.M., Armache, J.P., Berninghausen, O., Habeck, M., Subklewe, M., Wilson, D.N. and Beckmann, R. (2013) Structures of the human and Drosophila 80S ribosome. *Nature*, **497**, 80-85.

8. Shchepachev, V., Wischnewski, H., Missiaglia, E., Soneson, C. and Azzalin, C.M. (2012) Mpn1, mutated in poikiloderma with neutropenia protein 1, is a conserved 3'-to-5' RNA exonuclease processing U6 small nuclear RNA. *Cell Rep*, **2**, 855-865.

9. Mroczek, S., Krwawicz, J., Kutner, J., Lazniewski, M., Kucinski, I., Ginalski, K. and Dziembowski, A. (2012) C16orf57, a gene mutated in poikiloderma with neutropenia, encodes a putative phosphodiesterase responsible for the U6 snRNA 3' end modification. *Genes Dev*, **26**, 1911-1925.
